# Effects of Neurally Adjusted Ventilatory Assist (NAVA) levels in non-invasive ventilated patients: titrating NAVA levels with electric diaphragmatic activity and tidal volume matching

**DOI:** 10.1186/1475-925X-12-61

**Published:** 2013-07-02

**Authors:** Yeong Shiong Chiew, J Geoffrey Chase, Bernard Lambermont, Jean Roeseler, Christopher Pretty, Emilie Bialais, Thierry Sottiaux, Thomas Desaive

**Affiliations:** 1 Department of Mechanical Engineering, University of Canterbury, Christchurch, New Zealand; 2 GIGA Cardiovascular Science, University of Liege, Liege, Belgium; 3 Intensive Care Unit, Cliniques Universitaires St-Luc, Brussels, Belgium; 4 La clinique Notre Dame de Grâce, Gosselies, Belgium

**Keywords:** Mechanical ventilation, NAVA, Non-invasive ventilation, Patient-ventilator interaction, Matching

## Abstract

**Background:**

Neurally adjusted ventilatory assist (NAVA) delivers pressure in proportion to diaphragm electrical activity (*Eadi*). However, each patient responds differently to NAVA levels. This study aims to examine the matching between tidal volume (*Vt*) and patients’ inspiratory demand (*Eadi*), and to investigate patient-specific response to various NAVA levels in non-invasively ventilated patients.

**Methods:**

12 patients were ventilated non-invasively with NAVA using three different NAVA levels. NAVA100 was set according to the manufacturer’s recommendation to have similar peak airway pressure as during pressure support. NAVA level was then adjusted ±50% (NAVA50, NAVA150). Airway pressure, flow and *Eadi* were recorded for 15 minutes at each NAVA level. The matching of *Vt* and integral of *Eadi* (*ʃEadi*) were assessed at the different NAVA levels. A metric, Range90, was defined as the 5-95% range of *Vt*/*ʃEadi* ratio to assess matching for each NAVA level. Smaller Range90 values indicated better matching of supply to demand.

**Results:**

Patients ventilated at NAVA50 had the lowest Range90 with median 25.6 *uVs/ml* [Interquartile range (IQR): 15.4-70.4], suggesting that, globally, NAVA50 provided better matching between *ʃEadi* and *Vt* than NAVA100 and NAVA150. However, on a per-patient basis, 4 patients had the lowest Range90 values in NAVA100, 1 patient at NAVA150 and 7 patients at NAVA50. Robust coefficient of variation for *ʃEadi* and *Vt* were not different between NAVA levels.

**Conclusions:**

The patient-specific matching between *ʃEadi* and *Vt* was variable, indicating that to obtain the best possible matching, NAVA level setting should be patient specific. The Range90 concept presented to evaluate *Vt*/*ʃEadi* is a physiologic metric that could help in individual titration of NAVA level.

## Introduction

Non-invasive ventilation (NIV) is widely used in cases of acute respiratory failure [1] and for patients who are considered at risk of post-extubation respiratory failure [2]. However, NIV is usually delivered in pressure support (PS) mode despite the poor synchronization observed in intensive care unit (ICU) patients [3]. In comparison to PS, neurally adjusted ventilatory assist (NAVA) ventilation improves patient-ventilator interaction during invasive and NIV [4-7], and have shown to increase respiratory variability in comparison to PS [8].

NAVA triggers and cycles off the ventilator based on the patient’s diaphragmatic electrical activity (*Eadi*). The amount of pressure delivered by the ventilator is proportional to the *Eadi* amplitude [9]. Clinicians can adapt the amount of assist delivered with NAVA by selecting a NAVA level corresponding to a proportionality factor between instantaneously recorded *Eadi* and delivered pressure. Currently, there is limited information about how to correctly set patient-specific NAVA levels [10-14]. Additionally, implementing the best described method at the bedside [11] is difficult, potentially limiting the daily use of NAVA. The best way to adapt NAVA level on a day-to-day basis for individual patients is also unknown. Moreover, it is likely that each patient responds differently to various NAVA levels, complicating NAVA selection even further.

The aims of this study were two-fold. The primary goal was to investigate the matching between patient-specific inspiratory demand (*ʃEadi*) with ventilatory supply, tidal volume (*Vt*) at different NAVA levels during NIV. A second goal was the development of a new physiological approach for titrating NAVA level setting to the individual patient in a consistent fashion.

## Materials and methods

This study analyses *Eadi*-time, flow-time signal and derived parameters during NIV at three different NAVA levels. This study was conducted at the University Hospital of Liege (Liege, Belgium) and Cliniques Universitaires Saint-Luc (Brussels, Belgium). The Ethics Committees of both participating hospitals approved the study protocol and use of the data.

### Patients

The study cohort consisted of 12 non-invasively ventilated ICU patients. Patients were included in the study if they required NIV because of acute respiratory failure, or at risk of developing respiratory failure after extubation. Specific exclusion criteria were: 1) Severe hypoxemia requiring FiO_2_ > 0.6; 2) hemodynamic instability; 3) patient with a hiatal hernia or other oesophageal problem; upper gastrointestinal bleeding or any other contraindication to the insertion of a naso-gastric tube; 4) poor short term prognosis; and 5) age < 18 years. A summary of the patient demographic information with clinical diagnosis is shown in Table 1.

**Table 1:** Patient demographic information

**Patients**	**Gender**	**Age**	BMI*	SAPSII*	**Clinical diagnosis**
**1**	F	62	20.27	33	Exacerbation of COPD*
**2**	F	69	36.73	26	Pneumonia
**3**	F	56	16.7	33	Exacerbation of COPD
**4**	M	64	22.49	54	Acute renal failure
**5**	F	68	23.44	33	Asthma
**6**	M	75	26.12	34	Sepsis
**7**	M	87	23.67	28	Exacerbation of COPD
**8**	M	72	40.04	29	Exacerbation of COPD
**9**	M	87	27.68	30	Exacerbation of COPD
**10**	F	64	29.14	29	Cardiogenic pulmonary edema
**11**	F	79	27.97	30	Pneumonia
**12**	M	78	27.70	34	Drug intoxication
Median [IQR*]		70.5 [64.0-78.5]	26.9 [23.0-29.0]	31.5 [29.0-33.5]	

*COPD Chronic obstruct pulmonary disease, *IQR* Interquartile range, *BMI* Body mass index, *SAPSII* Simplified acute physiology score.

### Ventilator and delivered ventilation

All patients were ventilated with Servo-I ventilators (Maquet, Solna, Sweden) equipped with a commercially available NAVA module and software version 5.0. NIV was delivered through oronasal facemasks (Vygon SA, Ecouen, France) tightly attached in order to minimise the occurrence of leaks.

### Study protocol and recordings

After written informed consent was obtained, the patient’s standard nasogastric tube was replaced by NAVA tube. For each patient, 15 minutes of continuous recording (~200-300 breaths) was carried out at NAVA100. This NAVA100 level was set in order to have similar peak airway pressure (*P*_*in*_) as in PS mode using the previsualization system included with the ventilator. Two additional NAVA levels, denoted NAVA50 and NAVA150, that modified the initial NAVA level by ±50%, were used with an additional 15 minutes of breathing and continuous recordings.

*Eadi*, airway pressure and flow signals were acquired from the Servo-I ventilator, sampled at 100Hz using Servo-tracker V4.0 software (Maquet, Solna, Sweden). Positive end-expiratory pressure (PEEP), FiO_2_ and inspiratory trigger settings were maintained constant across each NAVA level for a given patient.

### Data analysis

#### Signal processing

The sign of the flow signal defined ventilator pressurisation. Pressurisation was defined to begin with a positive flow signal and end with a negative signal. Inspiratory tidal volume (*Vt*) for each breath was calculated by integrating the flow signal between the pressurisation beginning and end points. The length of time between these two points was termed the pressurisation time or inspiratory time (*Ti*). Breaths with *Vt* < 50 *ml* were discarded from analysis. This selection was made through post hoc analysis of the *Vt* distribution, suggesting that breaths with *Vt* < 50 *ml* likely corresponded to measurement artefacts.

The *Eadi* signal was integrated over the period *Ti* to obtain *ʃEadi*, representing patient inspiratory demand [8]. This approach did not account for the delay between the beginning of patient’s neural inspiration (reflected by the initial increase in *Eadi* signal) and the beginning of ventilator’s pressurization. However, this trigger delay is very low under NAVA [4], and did not significantly influence these results. By definition, the inspiration end point corresponded to the time when *Eadi* signal was decreased to 70% of the maximum *Eadi* signal as set in the NAVA software.

#### Range90 assessment of matching

**Figure 1:**
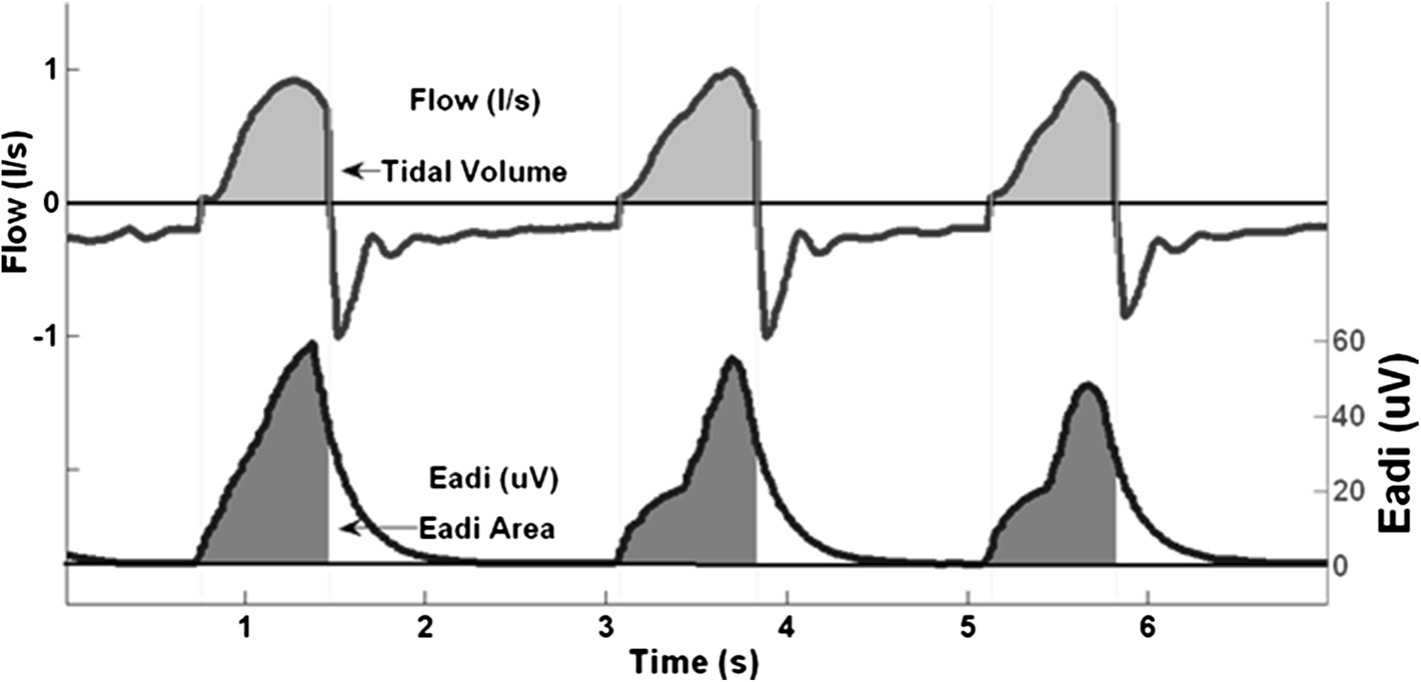
**Example of a patient’s inspiratory *ʃEadi *and resulting ventilatory *Vt*.**

In this study, *ʃEadi* was used to represent the intensity of the electrical activity during patient’s inspiration and thus, is the representation of the intensity of the patient’s inspiration effort. The resulting inspiratory *Vt*, corresponded to the supply delivered by the ventilator according to the patient’s demand. Figure 1 shows an example patient with demand, *ʃEadi* and corresponding ventilatory supply, *Vt*. Thus, *Vt*/*ʃEadi* is the ratio of outcome ventilator supply to patient demand (defined as Neuroventilatory efficiency [15]), and was assessed for each breath. The width of the 5-95^th^ percentile range of *Vt*/*ʃEadi* as shown in Equation (1) was calculated for each patient and NAVA level to enable analysis. This width was termed ‘Range90’ and defines a patient-specific metric characterizing the overall ‘*matching*’ between ventilator supply and patient demand.

(1) $$\mathrm{Range}90=95^{\mathrm{th}}\mathrm{Vt}/\int \mathrm{Eadi}-5^{\mathrm{th}}\mathrm{Vt}/\int \mathrm{Eadi}$$

If *Vt* for each breath were equally matched to the *ʃEadi*, then the *Vt*/*ʃEadi* ratio would be a constant. In contrast, a larger range of the *Vt*/*ʃEadi* ratio indicates an inability to consistently match *Vt* and *ʃEadi*. Thus, a smaller value of Range90 indicates consistently better matching of *Vt* to *ʃEadi*. Patients with larger values of Range90 have a higher incidence of inconsistent *Vt*/*ʃEadi* breaths, which is a lesser ability to match *Vt* and *ʃEadi,* regardless of the patient-specific *ʃEadi*. Matching, as captured by Range90, is thus the ability to match the variability of ventilator supply (*Vt*) to the variability of patient’s demand (*ʃEadi*).

Thus, the ratio of *Vt*/*ʃEadi* for each breath and the analysis of its distribution (Range90) over a given set of NAVA settings for a single patient enable a fair comparison between different NAVA levels. This simple metric could be calculated in real-time, for example by implementing dedicated software in the ventilator, to monitor patient-specific response to different NAVA levels. Hence, it may provide a simple solution to guide and titrate NAVA level. The detail and application of Range90 metric is reported elsewhere [16] and can also be found the Additional file 1 provided in the manuscript.

#### Statistical and correlation analysis

For each patient at each NAVA level, median [IQR (Interquartile range)] of *ʃEadi*, Peak inspiratory pressure (*P*_*in*_), *Vt*, and *Ti* were calculated. The distributions of *ʃEadi, P*_*in*_, *Vt* and *Ti* at 3 different NAVA levels were compared using the non-parametric Wilcoxon rank-sum test as they were not normally distributed. A Pearson’s correlation analysis was carried out for *Vt* with *ʃEadi* at different NAVA levels. Robust coefficient of variation (CVR = median absolute deviation/ median) was calculated for variability analysis in each parameter.

## Results

Table 2 summarizes patients’ *ʃEadi*, *P*_*in*_, *Vt*, and *Ti* at each NAVA level (median of medians with interquartile range [IQR]). Overall, NAVA50 had a higher *ʃEadi* = 18.5 *uVs* [IQR: 10.3-23.9] with lower *Vt* = 525 *ml* [IQR: 473–573] corresponding to a relatively low *P*_*in*_ = 15.9 *cmH*_*2*_*O* [IQR: 12.7-17.6]. In contrast, NAVA150 had lower *ʃEadi* (11.8 *uVs* [IQR: 7.8-17.9]) and higher *Vt* (630 *ml* [IQR: 478-721]) with *P*_*in*_ = 22.6 *cmH*_*2*_*O* [19.7-27.3]. *Ti* for NAVA50 was slightly higher at 0.88 s [IQR: 0.72-1.06] compared to other levels.

**Table 2:** Summary of inspiratory demand, peak inspiratory pressure, tidal volume and inspiratory time

	**Median of medians [IQR]**		
	**NAVA50**	**NAVA100**	**NAVA150**
**ʃ*Eadi *( *uVs *)**	18.5 [10.3-23.9]	13.7 [9.9-23.2]	11.8 [7.8-17.9]
***Peak Pressure, P***_***in ***_**(*cmH***_***2***_***O*)**	15.9 [12.7-17.6]	21.4 [16.9-22.9]*	22.6 [19.7-27.3]*
***Tidal Volume, Vt *( *ml *)**	525 [473-573]	581 [464-671]	630 [478-721]
***Inspiratory Time, Ti *(second)**	0.88 [0.72-1.06]	0.81 [0.68-1.03]	0.81 [0.68-1.01]

* P<0.05 compare to NAVA50.

Table 3 shows the Range90 values at different NAVA levels and Table 4 presents Pearson’s correlation coefficients between *ʃEadi* and the corresponding *Vt*. Comparing NAVA ventilation at different NAVA levels in Table 3, the Range90 value for the entire cohort was the smallest for NAVA50, with a median of 25.6 *uVs* [IQR: 15.4-70.4]. This result suggested that NAVA50 provided better matching for the cohort in general, compared to NAVA100 and NAVA150 where Range90 values were 31.7 *uVs* [IQR: 18.0-90.9] and 36.4 *uVs* [IQR: 18.9-109.8], respectively.

**Table 3:** Patients’ Range90 in different NAVA level

**Patient**	**Initial NAVA level**	**Range 90 (*Vt*/*ʃEadi*)**		
		**NAVA50**	**NAVA100**	**NAVA150**
**1**	0.2	14.7	8.5	14.0
**2**	0.6	93.7	38.4	138.6
**3**	0.2	36.3	16.0	17.1
**4**	0.8	48.6	110.9	80.9
**5**	0.4	20.5	84.0	40.2
**6**	0.6	20.5	22.9	32.5
**7**	0.8	7.2	24.9	68.0
**8**	0.4	10.8	12.4	17.4
**9**	0.4	16.1	19.9	20.4
**10**	1.0	30.7	47.2	30.5
**11**	1.0	277.9	97.7	207.2
**12**	0.5	92.2	141.8	211.9
**Median [IQR]**	0.55 [0.4-0.8]	25.6 [15.4-70.4]	31.7 [18.0-90.9]	36.4 [18.9-109.8]

**Table 4:** Pearson’s correlation

**Patients**	**Pearson’s correlation coefficient (*Vt*-*ʃEadi*)**		
	**NAVA50**	**NAVA100**	**NAVA150**
**1**	0.79	0.81	0.81
**2**	0.50	0.83	0.76
**3**	0.73	0.81	0.83
**4**	0.53	0.79	0.88
**5**	0.72	0.71	0.77
**6**	0.72	0.76	0.86
**7**	0.77	0.87	0.17
**8**	0.88	0.84	0.93
**9**	0.91	0.95	0.96
**10**	0.77	0.78	0.62
**11**	0.50	0.72	0.67
**12**	0.84	0.69	0.68
**Median [IQR]**	0.75 [0.63-0.82]	0.80 [0.74-0.84]	0.79 [0.68-0.87]

**Figure 2:**
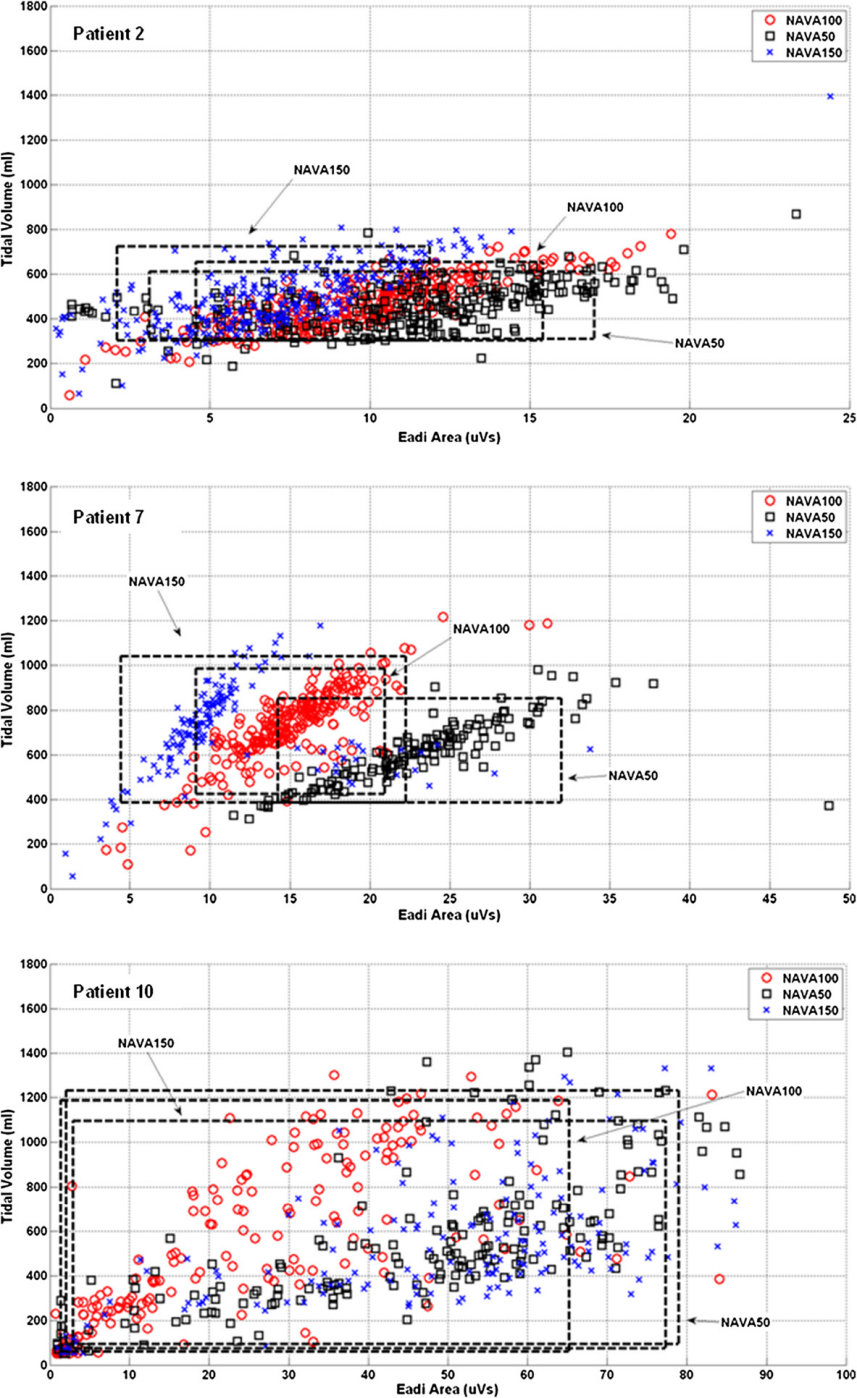
***Vt*-*ʃEadi *scatter plot.** (Top) Patient 2 - NAVA100, (Middle) Patient 7 - NAVA50, (Bottom) Patient 10 - NAVA150. The boxed areas show the breaths included in the 5-95^th^ range.

**Figure 3:**
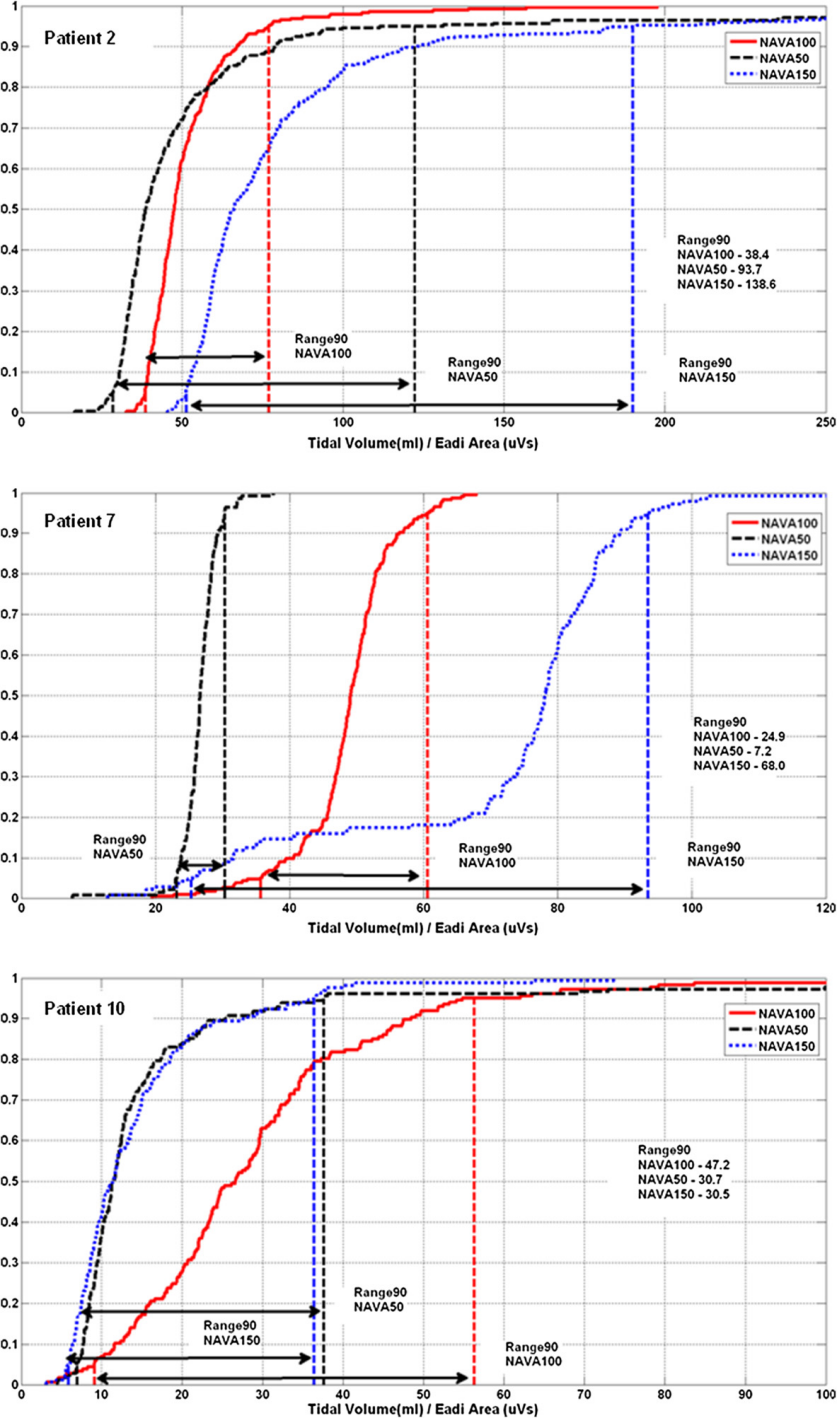
**Cumulative distribution for *Vt*/*ʃEadi *in Range90 analysis.** (Top) Patient 2 - NAVA100 has smaller Range90, (Middle) Patient 7 - NAVA 50 has smaller Range90, (Bottom) Patient 10 - NAVA150 and NAVA50 have a similar Range90, with NAVA150 smaller.

Figures 2 and 3 present the *Vt*-*ʃEadi* scatter plot and *Vt*/*ʃEadi* cumulative distribution plot for Patients 2, 7 and 10. These particular patients had minimum Range90 values (best matching) at each different NAVA level. Each Figure section highlights the patient-specific supply and demand for the specific NAVA level.

Table 5 shows the robust coefficients of variation for patients’ *ʃEadi*, *P*_*in*_, *Vt* and *Ti* at each NAVA level (p > 0.05 for all tested parameters). Further details relating to Tables 2 and 5 can be found in the Additional file 1 provided with the manuscript.

**Table 5:** Summary of robust coefficient of variation (CVR) inspiratory demand, peak inspiratory pressure, tidal volume and inspiratory time

	**CVR, median [IQR]**		
	**NAVA50**	**NAVA100**	**NAVA150**
***ʃEadi***	0.17 [0.13-0.31]	0.22 [0.14-0.31]	0.19 [0.15-0.32]
***Peak Pressure***	0.08 [0.06-0.12]	0.11 [0.08-0.14]	0.12 [0.07-0.18]
***Tidal Volume***	0.12 [0.08-0.19]	0.12 [0.09-0.28]	0.14 [0.09-0.24]
***Inspiratory Time***	0.08 [0.06-0.11]	0.07 [0.05-0.16]	0.09 [0.06-0.14]

## Discussion

Overall, these results (*ʃEadi*, *P*_*in*_, *Vt* and *Ti* trend) show that patients behaved in general as expected from other studies. More importantly, they also show that NAVA level was highly patient-specific due to significant inter-patient variability. The top and middle panes of Figure 2 (Patient 2 and 7) provide two examples where NAVA50 was associated with lower *Vt* and higher *ʃEadi* and NAVA150 was associated with higher *Vt* and lower *ʃEadi*. The results for NAVA100 were located between those of NAVA50 and NAVA150.

Schmidt et al. [8] and Patroniti et al. [17] showed that a higher NAVA level resulted in a lower *Eadi* magnitude with higher *Vt*. Higher NAVA level delivers higher pressure, proportional to the level settings, possibly resulting in higher ventilator supply, *Vt*. Thus, the *Eadi* signal that represents the patient-specific demand may decrease. Hence, the overall results in our study match other published results [7,11,13,17-19].

No significant intra-patient difference between NAVA levels was found in the variability of *ʃEadi*, *P*_*in*_, *Vt*, and *Ti*. This result indicates that ventilation at different NAVA levels results in similar variability [7,11,13,17-19]. Thus, selecting an optimal NAVA level for a patient based on variability analysis is not suitable.

NAVA levels influence the overall matching (Range90) between patient inspiratory demand and delivered *Vt*. Overall, NAVA50 gave the best matching (lowest Range90) for the entire cohort. However, cohort results can be misleading for individual patients. More specifically, Patient 10 had a minimum Range90 value at NAVA150, 4 patients had minimum value at NAVA100 (Patients 1-3, 11) and 7 patients (Patients 4-9, 12) at NAVA50. These results show the clear inter-patient variability, and the perils of only considering cohort-based measures.

The results show the clinical potential of using *Vt*/*ʃEadi* and the Range90 metric to titrate patient-specific NAVA level over a heterogeneous patient cohort to obtain the best possible *Vt*/*ʃEadi* matching. Practically, such an approach could be used in real time if it was implemented in the ventilator to choose the best NAVA level for a given patient at a given time. This approach could also prove useful for adapting NAVA level over time, during a patient’s stay, and especially during weaning from mechanical ventilation, where only limited data are currently available [10].

It was observed that several patients had very similar values of Range90 in two different NAVA levels. Three patients with only ±10% difference were Patient 3 (NAVA100 and NAVA150), Patient 6 (NAVA50 and NAVA150) and Patient 10 (NAVA50 and NAVA150). These results indicate that the effect of the different NAVA levels were less significant in the matching of ventilator supply and patient demand. This finding also indicates that NAVA level titration through interpolation of Range90 would not be effective as the supply and demand matching does not correspond linearly to NAVA level. Similarly, titrating NAVA towards higher or lower levels in some cases (Patients 6 and 10) may be beneficial in terms of ventilator supply and demand matching.

Figures 2 and 3 show *Vt*/*ʃ*Eadi cumulative distribution and *Vt*-*ʃ*Eadi plots for Patient 2, 7 and 10 with Range90 values at each NAVA level. These 3 patients had minimum Range90 values at different NAVA levels. More specifically, the Range90 metric suggested that Patient 2 should be ventilated at the original NAVA100 level, Patient 7 could have the original NAVA level of 0.80 reduced by 50% for better matching, and Patient 10 would be better matched at the higher NAVA150 level.

Correlation coefficients facilitate examination of the relationship between *Vt* with *ʃEadi*, independent of the effects of NAVA level on the magnitude of *Eadi* signals. The correlation coefficient between *Vt* and *ʃEadi* may potentially be another metric to aid in titrating NAVA level. The correlation coefficients for different NAVA levels in this study were similar, indicating that NAVA was able to consistently match supply with demand at different levels. However, individual patients showed otherwise. For example, Patient 2 (NAVA50), Patient 4 (NAVA50), Patient 7 (NAVA150) and Patient 11 (NAVA50), showed significantly lower *R* values compared to other NAVA levels, indicating significant supply and demand mismatch at a specific patient level. Equally, the small changes in the value of *R* between NAVA levels may not be clinically significant, indicating that correlation coefficient was not as sensitive to changes in NAVA level as the Range90 metric. The Range90 metric consistently identified *Vt*/*ʃEadi* mismatch between NAVA levels compared to Pearson’s correlation, yielding a potentially more sensitive metric.

## Limitations

Several potential limitations of this study must be pointed out. First, NAVA100 was defined to match the value of peak airway pressure during PS as set by clinicians. However, there was no standardisation of PS settings, and the appropriate level of assistance remains debated.

This study was conducted during NIV ventilation. During NIV, leaks can occur at the patient-mask interface and can influence delivered *Vt*. However, for this study, the mask was tightly attached to the patient by an experienced therapist in order to minimize the chance of leaks. Additionally, the therapist remained at the bedside during the whole recording to adapt the mask if necessary. These precautions made major leaks at the patient mask interface very unlikely.

Only 3 levels of NAVA were explored for each patient, separated by ±50% from the original NAVA100 level. At ±50%, the absolute changes of NAVA level can be very small or large depending on initial NAVA level. One consequence of such widely spaced NAVA levels is that, potentially none of the 3 tested NAVA levels in the trials were optimal. Thus, a more refined set of NAVA levels might well show a better result with this metric at a different NAVA level.

Finally, it is important to note that while the Range90 metric showed better matching for specific NAVA levels, the advantage of using this specific NAVA level is not yet clinically proven. Prior work has shown better matching of *Vt* to *ʃ*Eadi demand results in less asynchrony in comparing NAVA ventilation and PS [20]. However, the use of Range90 to titrate NAVA levels for better physiological outcome remains to be prospectively tested and the results here show only the sensitivity to different level settings and inter-patient variability thus demonstrating the potential clinical interest.

## Conclusions

Based on matching and correlation analysis, it was found that each patient reacted differently to different NAVA levels. This finding indicates significant inter-patient variability and patient-specific response. Using the proposed concept of supply and demand ratios (Range90), more optimal NAVA levels can be found and titrated for each patient based on the simple Range90 metric. This approach can later be used in real time to adapt NAVA levels if included in software.

## Abbreviations

CVR: Robust coefficient of variation; Eadi: Diaphragm electrical activity; ʃEadi: Patient inspiratory demand (Eadi Area); FiO2: Fraction of inspired oxygen; ICU: Intensive care unit; IQR: Interquartile range; NAVA: Neurally adjusted ventilatory assist; NIV: Non invasive ventilation; PEEP: Positive end expiratory pressure; Pin: Peak inspiratory pressure; PS: Pressure support; Ti: Pressurisation time or inspiratory time; Vt: Tidal volume; BMI: Body mass index; COPD: Chronic obstruct pulmonary disease; SAPSII: Simplified acute physiology score.

## Competing interest

The authors declared that they have no competing interest.

## Authors’ contributions

YSC, TD, CP, and JGC defined the metric. All authors had input to analysis of results. BL, JR and EB implemented trials clinically with input from all others. All authors had input in writing and revising the manuscript.

## Supplementary Material

Additional file 1 Effects of Various Neurally Adjusted Ventilatory Assist (NAVA) levels on the matching between electric diaphragmatic activity and tidal volume. Click here for file
