# Supplementary material for: Effects of Neurally Adjusted Ventilatory Assist (NAVA) levels in non-invasive ventilated patients: titrating NAVA levels with electric diaphragmatic activity and tidal volume matching
Source: Biomed Eng Online. 2013 Jul 2;12:61. doi: 10.1186/1475-925X-12-61 (PMC3707774; doi:10.1186/1475-925X-12-61)
Supplement: Additional file 1 — Effects of Various Neurally Adjusted Ventilatory Assist (NAVA) levels on the matching between electric diaphragmatic activity and tidal volume. [file 1475-925X-12-61-S1.doc]

**Online Supplement for**

**Effects of Various Neurally Adjusted Ventilatory Assist (NAVA) Levels on the Matching between Electric Diaphragmatic Activity and Tidal Volume**

The following shows the method for Range90 calculation in a patient:

1. Obtain tidal volume (*Vt*) and *ʃEadi* for each breathing cycle as shown in Figures 1 and 2.
2. The Neuroventilatory efficiency (*Vt*/*ʃEadi* ratio) for each breathing cycle is calculated.

(Example: *Vt*_1_*/ʃEadi*_1_, *Vt*_2_*/ʃEadi*_2_, *Vt*_3_*/ʃEadi*_3_, *Vt*_4_*/* *ʃEadi*_4_ …, *Vt*_n_*/ʃEadi*_n_).

1. The 5^th^ percentile of every *Vt*/*ʃEadi* ratio is determined (5^th^ *Vt/ʃEadi*).
2. The 95^th^ percentile of every *Vt*/*ʃEadi* ratio is determined (95^th^ *Vt/ʃEadi*).
3. Range90 = 95^th^ *Vt*/*ʃEadi* - 5^th^ *Vt*/*ʃEadi*
4. For a patient who has consistent *Vt*/*ʃEadi* ratio, Range90 will be smaller.
5. For a patient who has variable *Vt*/*ʃEadi* ratio, Range90 will be higher.


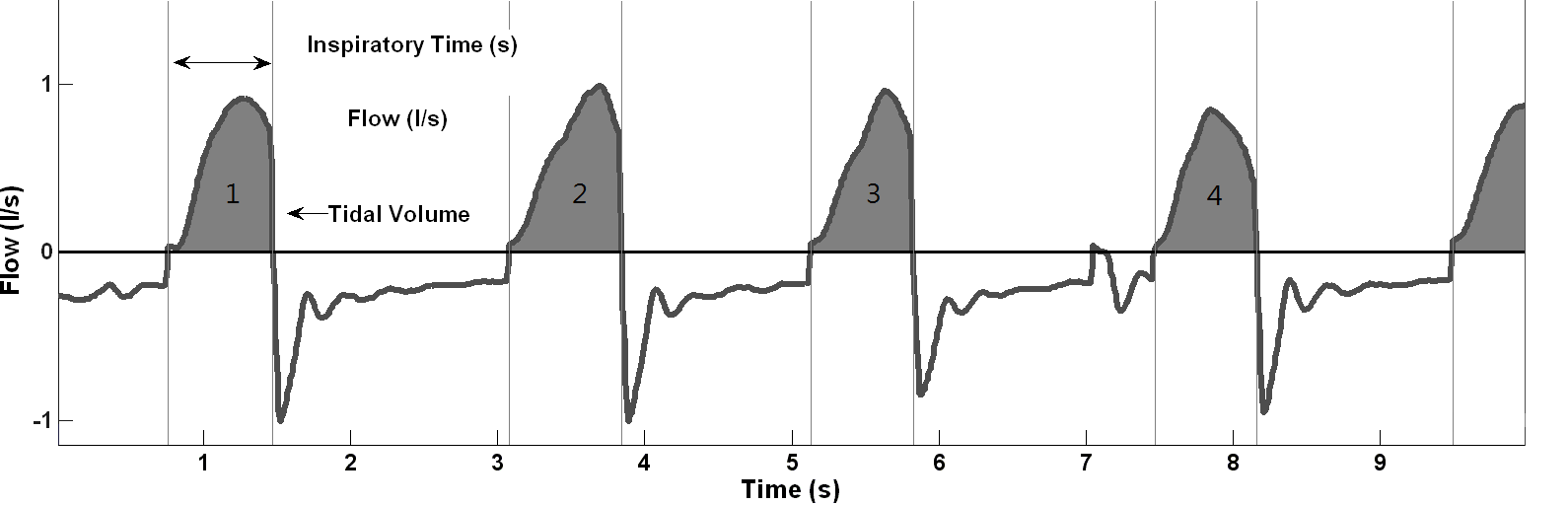


Figure 1: Example of a patient’s flow-time curve. The shaded area is the ventilator supply (Tidal volume, *Vt*)


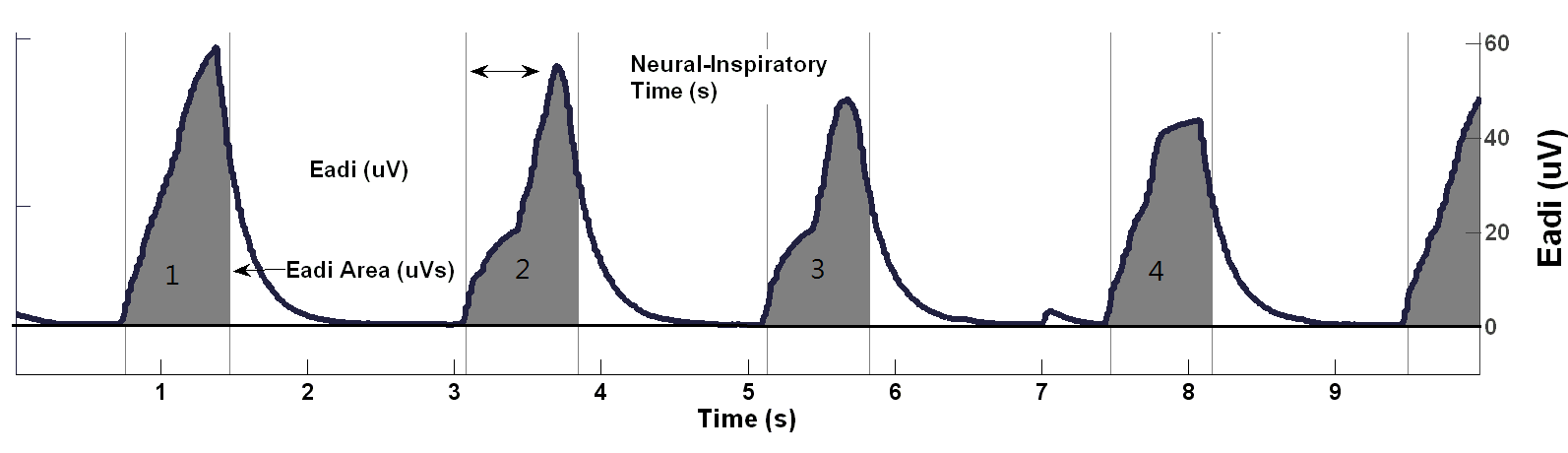


Figure 2: Example of a patient’s *Eadi*-time curve. The shaded area is the patient’s demand (*ʃEadi*)

**Case example: Comparing better and less matching**

Example: *Vt* = 1.0 and *ʃEadi* = 1.0 for baseline value

**Case A - Patient with better matching**

*ʃEadi* = 1.0, *Vt* = 1.0, *Vt*/*ʃEadi* = 1.0 (Moderate demand, Moderate supply)

*ʃEadi* = 2.0, *Vt* = 2.0, *Vt*/*ʃEadi* = 1.0 (High demand, High supply)

*ʃEadi* = 0.5, *Vt* = 0.5, *Vt*/*ʃEadi* = 1.0 (Low demand, Low supply)

…

95^th^-5^th^ of all *Vt*/ ʃ*Eadi* for Case A will be small resulting in smaller Range90 value.

**Case B - Patient with less matching**

*ʃEadi* = 1.0, *Vt* = 2.0, *Vt*/*ʃEadi* = 2.0 (Moderate demand, High supply)

*ʃEadi* = 2.0, *Vt* = 1.0, *Vt*/*ʃEadi* = 0.5 (High demand, Moderate supply)

*ʃEadi* = 0.5, *Vt* = 1.0, *Vt*/*ʃEadi* = 1.0 (Low demand, Moderate supply)

…

Thus, 95^th^-5^th^ of all *Vt*/*ʃEadi* for Case B will be higher, with larger Range90 value compared to Case A. Case A has better matching than Case B.

**Case examples: Higher Variability may not be better matching**

**Case C – (High *ʃEadi* variability, High *Vt* variability) Patient with less matching**

*ʃEadi* = 1.0, *Vt* = 3.0, *Vt/ʃEadi* = 3.0 (Moderate demand, High supply)

*ʃEadi* = 2.0, *Vt* = 1.0, *Vt/ʃEadi* = 0.5 (High demand, Moderate supply)

*ʃEadi* = 0.5, *Vt* = 2.0, *Vt/ʃEadi* = 1.0 (Low demand, Moderate supply)

…

**Case D – (Low *ʃEadi* variability, Low *Vt* variability) Patient with better matching**

*ʃEadi* = 1.0, *Vt* = 1.0, *Vt/ʃEadi* = 1.0 (Moderate demand, High supply)

*ʃEadi* = 1.0, *Vt* = 1.0, *Vt/ʃEadi* = 1.0 (High demand, Moderate supply)

*ʃEadi* = 1.0, *Vt* = 1.0, *Vt/ʃEadi* = 1.0 (Low demand, Moderate supply)

…

While Case D had low *ʃEadi* and *Vt* variability than Case C, it has better matching.

**Additional Results and Findings**

e-Table 1-7 present patients’ inspiratory demand *ʃEadi*, peak inspiratory pressure (*P_in_*), ventilatory tidal volume (*Vt*), pressurisation time (*Ti*), leaks (*Vt_leaks*) (difference between inspiration and expiration tidal volume), duty cycle (Pressurisation time/ Total inspiration and expiration time, *Ti/Ttot*) and Neural Inspiratory time (Time from start of flow to maximum Eadi signal occurs, *Ti_Neural*) at each NAVA level, as summarised in the manuscript. Wilcoxon rank sum test for significance was carried out in every patient to compare the effect of different NAVA level.

e-Table 8-12 present patients’ robust coefficient of variation (CVR) on inspiratory demand *ʃEadi*, peak inspiratory pressure (*P_in_*), ventilatory tidal volume (*Vt*), inspiratory time (*Ti*) and Neural Inspiratory time (*Ti_Neural*) each NAVA level, as summarised in the manuscript. Wilcoxon rank sum test for significance was carried out in every patient to compare the effect of different NAVA level.

**e-Table 1:** Patient’s inspiratory demand (*ʃEadi*)

| **Patient** | ***ʃEadi* (*uVs*), Median [IQR]** | | |
| --- | --- | --- | --- |
|  | **NAVA50** | **NAVA100** | **NAVA150** |
| **1** | 24.5 [21.2-27.6] | 23.7 [21.2-26.5] | 19.1 [16.4-22.4]*+ |
| **2** | 11.3 [8.1-13.9] | 9.9 [7.8-11.7]* | 6.9 [5.1-9.0]*+ |
| **3** | 22.6 [17.1-25.6] | 22.7 [19.5-25.7] | 21.2 [18.3-23.8]+ |
| **4** | 10.1 [8.7-11.5] | 8.0 [6.5-10.4]* | 8.6 [5.6-10.3]* |
| **5** | 15.8 [14.5-17.7] | 13.9 [10.4-16.1]* | 15.2 [12.9-17.3]*+ |
| **6** | 10.5 [9.8-11.4] | 9.8 [8.9-10.7]* | 9.0 [8.1-9.8]*+ |
| **7** | 23.3 [19.8-25.9] | 15.0 [13.1-17.4]* | 9.9 [8.3-11.7]*+ |
| **8** | 31.5 [26.3-36.3] | 41.1 [31.2-51.3]* | 16.6 [14.0-19.1]*+ |
| **9** | 21.1 [15.9-28.6] | 13.5 [9.5-18.8]* | 13.7 [9.3-18.9]* |
| **10** | 50.9 [31.5-61.9] | 24.3 [7.6-39.9]* | 54.9 [36.0-62.9]+ |
| **11** | 8.3 [5.2-11.6] | 10.1 [7.5-13.4]* | 5.9 [4.0-8.7]*+ |
| **12** | 4.2 [2.4-7.7] | 6.7 [3.3-10.7]* | 5.3 [2.6-8.5]+ |
| **Median of Medians [IQR]** | 18.5 [10.3-23.9] | 13.7 [9.9-23.2] | 11.8 [7.8-17.9] |

** P<0.05 compare to NAVA50*

*+ P<0.05 compare to NAVA100*

**e-Table 2: Peak inspiratory pressure (*P_in_*)**

| **Patient** | **Peak Pressure (*cmH_2_O*), Median [IQR]** | | |
| --- | --- | --- | --- |
|  | **NAVA50** | **NAVA100** | **NAVA150** |
| **1** | 11.5 [10.6-12.6] | 16.4 [15.3-17.9]* | 18.7 [17.2-20.7]*+ |
| **2** | 17.1 [14.9-19.2] | 21.3 [18.9-24.3]* | 23.3 [19.8-27.0]*+ |
| **3** | 12.1 [11.4-12.9] | 17.3 [16.2-18.5]* | 21.6 [20.2-23.4]*+ |
| **4** | 14.4 [13.1-15.4] | 19.8 [17.2-22.6]* | 25.4 [20.7-28.6]*+ |
| **5** | 20.1 [19.1-21.1] | 27.3 [25.1-29.5]* | 35.2 [33.4-35.7]*+ |
| **6** | 16.9 [16.1-17.9] | 23.2 [21.9-24.5]* | 28.1 [26.3-30.3]*+ |
| **7** | 20.2 [17.4-23.3] | 25.0 [22.2-27.9]* | 26.4 [21.1-31.0]*+ |
| **8** | 16.2 [14.7-17.0] | 22.5 [19.9-24.9]* | 21.8 [20.4-24.7]* |
| **9** | 18.1 [15.9-19.9] | 21.5 [17.5-24.0]* | 28.4 [22.4-33.0]*+ |
| **10** | 13.2 [12.9-13.8] | 13.2 [11.8-14.4] | 11.5 [10.7-11.8]*+ |
| **11** | 15.5 [12.9-18.9] | 22.3 [18.7-25.9]* | 20.6 [17.1-25.8]*+ |
| **12** | 5.9 [5.6-6.6] | 8.1 [6.7-9.5]* | 7.1 [5.9-8.3]*+ |
| **Median of Medians [IQR]** | 15.9 [12.7-17.6] | 21.4 [16.9-22.9]* | 22.6 [19.7-27.3]* |

** P<0.05 compare to NAVA50*

*+ P<0.05 compare to NAVA100*

**e-Table 3: Ventilatory tidal volume (*Vt*)**

| **Patient** | **Tidal Volume*, Vt* (*ml*), Median [IQR]** | | |
| --- | --- | --- | --- |
|  | **NAVA50** | **NAVA100** | **NAVA150** |
| **1** | 526 [435-564] | 444 [404-490]* | 474 [424-541]*+ |
| **2** | 450 [379-524] | 471 [389-544] | 481 [399-586]* |
| **3** | 324 [294-345] | 372 [339-406]* | 416 [379-450]*+ |
| **4** | 654 [597-698] | 738 [671-810]* | 788 [584-895]* |
| **5** | 549 [525-578] | 631 [594-675]* | 727 [679-764]*+ |
| **6** | 596 [574-621] | 688 [657-718]* | 763 [725-802]*+ |
| **7** | 608 [518-689] | 741 [653-833]* | 715 [605-836]* |
| **8** | 522 [468-559] | 646 [451-862]* | 621 [544-696]* |
| **9** | 523 [411-660] | 531 [404-657] | 664 [466-854]*+ |
| **10** | 496 [343-705] | 487 [244-908] | 485 [354-689] |
| **11** | 543 [486-610] | 653 [585-726]* | 638 [553-688]*+ |
| **12** | 270 [179-391] | 457 [250-613]* | 413 [208-708]* |
| **Median of Medians [IQR]** | 525 [473-573] | 581 [464-671] | 630 [478-721] |

** P<0.05 compare to NAVA50*

*+ P<0.05 compare to NAVA100*

**e-Table 4: Pressurisation time (*Ti*)**

| **Patient** | **Pressurisation time*, Ti* (second), Median [IQR]** | | |
| --- | --- | --- | --- |
|  | **NAVA50** | **NAVA100** | **NAVA150** |
| **1** | 0.86 [0.77-0.95] | 0.72 [0.70-0.78]* | 0.73 [0.69-0.77]* |
| **2** | 0.78 [0.73-0.83] | 0.78 [0.72-0.84] | 0.74 [0.68-0.81]*+ |
| **3** | 0.70 [0.64-0.74] | 0.69 [0.63-0.73] | 0.70 [0.65-0.75] |
| **4** | 0.90 [0.87-0.94] | 0.84 [0.80-0.89]* | 0.87 [0.77-0.96]* |
| **5** | 0.55 [0.51-0.57] | 0.54 [0.50-0.57] | 0.55 [0.51-0.57] |
| **6** | 0.65 [0.63-0.67] | 0.65 [0.62-0.67] | 0.65 [0.62-0.67] |
| **7** | 1.10 [1.00-1.20] | 0.98 [0.91-1.05]* | 0.96 [0.86-1.02]* |
| **8** | 1.78 [1.58-1.94] | 1.89 [1.60-2.10] | 1.57 [1.35-1.80]*+ |
| **9** | 0.73 [0.63-0.89] | 0.67 [0.55-0.80]* | 0.62 [0.53-0.81]* |
| **10** | 1.55 [1.35-1.71] | 1.15 [0.74-1.44]* | 1.55 [1.28-1.71]+ |
| **11** | 1.01 [0.94-1.06] | 1.08 [0.98-1.15]* | 1.06 [0.99-1.12]* |
| **12** | 0.98 [0.82-1.12] | 0.87 [0.69-1.05]* | 0.93 [0.75-1.15]+ |
| **Median of Medians [IQR]** | 0.88 [0.72-1.06] | 0.81 [0.68-1.03] | 0.81 [0.68-1.01] |

** P<0.05 compare to NAVA50*

*+ P<0.05 compare to NAVA100*

**e-Table 5: Leaks (*Vt_leak*)**

| **Patient** | **Leak, *Vt_leak* (*ml*), Median [IQR]** | | |
| --- | --- | --- | --- |
|  | **NAVA50** | **NAVA100** | **NAVA150** |
| **1** | 112 [48-211] | 56 [29-97]* | 65 [35-116]*+ |
| **2** | 55 [37-76] | 75 [49-109]* | 66 [42-89]*+ |
| **3** | 50 [28-130] | 44 [22-73]* | 44 [24-78]* |
| **4** | 201 [161-258] | 217 [182-270]* | 346 [242-455]* |
| **5** | 84 [71-101] | 79 [59-100]* | 100 [79-120]*+ |
| **6** | 28 [13-45] | 38 [25-51]* | 43 [29-59]*+ |
| **7** | 66 [34-173] | 94 [49-284]* | 103 [40-305]* |
| **8** | 35 [18-72] | 135 [66-231]* | 57 [20-93]+ |
| **9** | 49 [22-88] | 45 [22-83] | 81 [37-147]*+ |
| **10** | 141 [54-273] | 177 [61-312] | 95 [43-178]*+ |
| **11** | 43 [18-126] | 40 [16-85] | 49 [23-86]+ |
| **12** | 134 [34-274] | 181 [78-407]* | 197 [83-509]* |
| **Median of Medians [IQR]** | 61 [46-123] | 77 [45-156] | 74 [53-102] |

** P<0.05 compare to NAVA50*

*+ P<0.05 compare to NAVA100*

**e-Table 6: Duty cycle (*Ti/Ttot*)**

| **Patient** | **Duty Cycle (*Ti/Ttot*), Median [IQR]** | | |
| --- | --- | --- | --- |
|  | **NAVA50** | **NAVA100** | **NAVA150** |
| **1** | 0.35 [0.27-0.48] | 0.35 [0.33-0.37] | 0.34 [0.30-0.36]*+ |
| **2** | 0.41 [0.39-0.44] | 0.40 [0.38-0.43]* | 0.39 [0.36-0.41]*+ |
| **3** | 0.32 [0.30-0.37] | 0.31 [0.29-0.34]* | 0.30 [0.29-0.33]*+ |
| **4** | 0.42 [0.40-0.43] | 0.40 [0.39-0.42]* | 0.42 [0.40-0.71]+ |
| **5** | 0.29 [0.28-0.31] | 0.29 [0.27-0.31]* | 0.31 [0.29-0.32]*+ |
| **6** | 0.40 [0.38-0.41] | 0.36 [0.35-0.38]* | 0.37 [0.35-0.38]*+ |
| **7** | 0.40 [0.36-0.52] | 0.41 [0.36-0.59] | 0.36 [0.32-0.59]*+ |
| **8** | 0.40 [0.37-0.46] | 0.39 [0.35-0.45] | 0.37 [0.32-0.41]*+ |
| **9** | 0.37 [0.29-0.43] | 0.38 [0.31-0.43] | 0.38 [0.33-0.42] |
| **10** | 0.35 [0.31-0.48] | 0.41 [0.31-0.70] | 0.33 [0.30-0.41]*+ |
| **11** | 0.40 [0.38-0.43] | 0.39 [0.37-0.41]* | 0.39 [0.37-0.42]* |
| **12** | 0.43 [0.32-0.96] | 0.33 [0.25-0.77]* | 0.33 [0.26-0.77]* |
| **Median of Medians [IQR]** | 0.40 [0.35-0.41] | 0.39 [0.34-0.40] | 0.37 [0.33-0.39] |

** P<0.05 compare to NAVA50*

*+ P<0.05 compare to NAVA100*

**e-Table 7: Neural inspiratory time (*Ti*)**

| **Patient** | **Neural inspiratory time*, Ti_Neural* (second), Median [IQR]** | | |
| --- | --- | --- | --- |
|  | **NAVA50** | **NAVA100** | **NAVA150** |
| **1** | 0.68 [0.61-0.75] | 0.62 [0.56-0.65]* | 0.61 [0.54-0.65]*+ |
| **2** | 0.64 [0.58-0.68] | 0.62 [0.57-0.68] | 0.59 [0.53-0.66]*+ |
| **3** | 0.54 [0.48-0.59] | 0.52 [0.48-0.59] | 0.53 [0.49-0.60] |
| **4** | 0.78 [0.74-0.82] | 0.72 [0.68-0.77]* | 0.74 [0.62-0.82]* |
| **5** | 0.40 [0.36-0.43] | 0.40 [0.36-0.44] | 0.40 [0.36-0.44] |
| **6** | 0.52 [0.48-0.56] | 0.51 [0.47-0.55]* | 0.50 [0.46-0.56]* |
| **7** | 0.94 [0.85-1.04] | 0.81 [0.72-0.88]* | 0.77 [0.66-0.85]*+ |
| **8** | 1.63 [1.39-1.76] | 1.65 [1.33-1.91] | 1.25 [1.08-1.48]*+ |
| **9** | 0.58 [0.48-0.73] | 0.51 [0.41-0.65]* | 0.49 [0.40-0.64]* |
| **10** | 1.32 [0.98-1.52] | 0.92 [0.53-1.23]* | 1.27 [1.03-1.49]+ |
| **11** | 0.86 [0.78-0.92] | 0.90 [0.80-1.00]* | 0.90 [0.80-0.98]* |
| **12** | 0.77 [0.59-0.93] | 0.64 [0.50-0.82]* | 0.70 [0.53-0.89]+ |
| **Median of Medians [IQR]** | 0.73 [0.56-0.90] | 0.63 [0.52-0.86] | 0.66 [0.52-0.84] |

** P<0.05 compare to NAVA50*

*+ P<0.05 compare to NAVA100*

**e-Table 8:** Robust coefficient of variation (CVR) for patient’s inspiratory demand (*ʃEadi*)

| **Patient** | **NAVA50** | **NAVA100** | **NAVA150** |
| --- | --- | --- | --- |
| **1** | 0.13 | 0.11 | 0.16 |
| **2** | 0.24 | 0.20 | 0.29 |
| **3** | 0.18 | 0.14 | 0.12 |
| **4** | 0.13 | 0.26 | 0.27 |
| **5** | 0.10 | 0.19 | 0.15 |
| **6** | 0.08 | 0.09 | 0.09 |
| **7** | 0.14 | 0.14 | 0.17 |
| **8** | 0.16 | 0.25 | 0.15 |
| **9** | 0.36 | 0.33 | 0.35 |
| **10** | 0.29 | 0.68 | 0.21 |
| **11** | 0.39 | 0.30 | 0.38 |
| **12** | 0.52 | 0.54 | 0.55 |
| **Median [IQR]** | 0.17 [0.13-0.33] | 0.23 [0.14-0.32] | 0.19 [0.15-0.32] |

**e-Table 9:** Robust coefficient of variation (CVR) for peak inspiratory pressure (*P_in_*)

| **Patient** | **NAVA50** | **NAVA100** | **NAVA150** |
| --- | --- | --- | --- |
| **1** | 0.09 | 0.08 | 0.09 |
| **2** | 0.13 | 0.12 | 0.16 |
| **3** | 0.06 | 0.07 | 0.08 |
| **4** | 0.08 | 0.13 | 0.16 |
| **5** | 0.05 | 0.08 | 0.01 |
| **6** | 0.05 | 0.06 | 0.07 |
| **7** | 0.15 | 0.11 | 0.19 |
| **8** | 0.08 | 0.10 | 0.09 |
| **9** | 0.11 | 0.14 | 0.18 |
| **10** | 0.03 | 0.10 | 0.03 |
| **11** | 0.19 | 0.16 | 0.23 |
| **12** | 0.08 | 0.17 | 0.17 |
| **Median [IQR]** | 0.08 [0.06-0.12] | 0.11 [0.08-0.14] | 0.13 [0.08-0.17] |

**e-Table 10:** Robust coefficient of variation (CVR) for ventilatory tidal volume (*Vt*)

| **Patient** | **NAVA50** | **NAVA100** | **NAVA150** |
| --- | --- | --- | --- |
| **1** | 0.12 | 0.09 | 0.12 |
| **2** | 0.16 | 0.17 | 0.19 |
| **3** | 0.09 | 0.09 | 0.09 |
| **4** | 0.07 | 0.09 | 0.17 |
| **5** | 0.05 | 0.07 | 0.06 |
| **6** | 0.04 | 0.05 | 0.05 |
| **7** | 0.14 | 0.12 | 0.16 |
| **8** | 0.09 | 0.32 | 0.12 |
| **9** | 0.23 | 0.24 | 0.30 |
| **10** | 0.34 | 0.63 | 0.34 |
| **11** | 0.12 | 0.11 | 0.10 |
| **12** | 0.35 | 0.39 | 0.54 |
| **Median [IQR]** | 0.12 [0.08-0.20] | 0.12 [0.09-0.28] | 0.14 [0.10-0.25] |

**e-Table 11:** Robust coefficient of variation (CVR) for inspiratory time (*Ti*)

| **Patient** | **NAVA50** | **NAVA100** | **NAVA150** |
| --- | --- | --- | --- |
| **1** | 0.10 | 0.04 | 0.05 |
| **2** | 0.06 | 0.08 | 0.09 |
| **3** | 0.07 | 0.07 | 0.07 |
| **4** | 0.04 | 0.05 | 0.10 |
| **5** | 0.05 | 0.06 | 0.05 |
| **6** | 0.03 | 0.03 | 0.03 |
| **7** | 0.09 | 0.07 | 0.08 |
| **8** | 0.10 | 0.12 | 0.15 |
| **9** | 0.17 | 0.19 | 0.18 |
| **10** | 0.12 | 0.32 | 0.13 |
| **11** | 0.06 | 0.08 | 0.07 |
| **12** | 0.16 | 0.21 | 0.20 |
| **Median [IQR]** | 0.08 [0.06-0.11] | 0.08 [0.06-0.16] | 0.09 [0.06-0.14] |

**e-Table 12:** Robust coefficient of variation (CVR) for neural inspiratory time (*Ti_Neural*)

| **Patient** | **NAVA50** | **NAVA100** | **NAVA150** |
| --- | --- | --- | --- |
| **1** | 0.10 | 0.05 | 0.08 |
| **2** | 0.08 | 0.10 | 0.10 |
| **3** | 0.09 | 0.10 | 0.09 |
| **4** | 0.05 | 0.06 | 0.14 |
| **5** | 0.10 | 0.10 | 0.10 |
| **6** | 0.08 | 0.07 | 0.10 |
| **7** | 0.10 | 0.10 | 0.13 |
| **8** | 0.10 | 0.18 | 0.17 |
| **9** | 0.21 | 0.24 | 0.22 |
| **10** | 0.19 | 0.38 | 0.17 |
| **11** | 0.08 | 0.11 | 0.10 |
| **12** | 0.21 | 0.25 | 0.26 |
| **Median [IQR]** | 0.10 [0.08-0.15] | 0.10 [0.09-0.21] | 0.12 [0.10-0.17] |

Figures below show the cumulative distribution plot for *Vt/ ʃEadi* in all patients at different NAVA levels. NAVA100 red), NAVA50 (black), NAVA 150 (blue) and the 5^th^, 50^th^ and 95^th^ percentile lines.

**Patient 1**

**
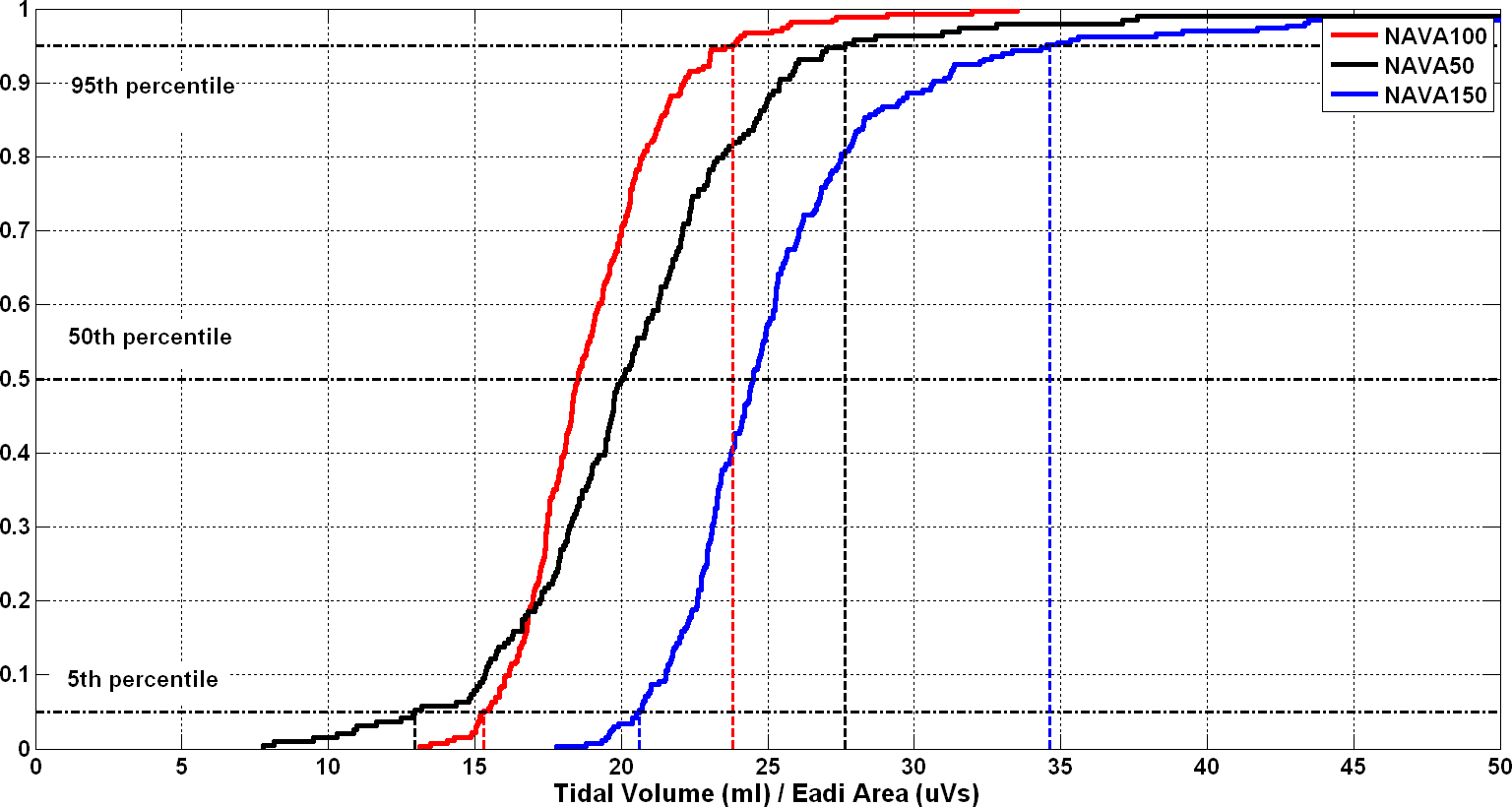
**

**Patient 2**

**
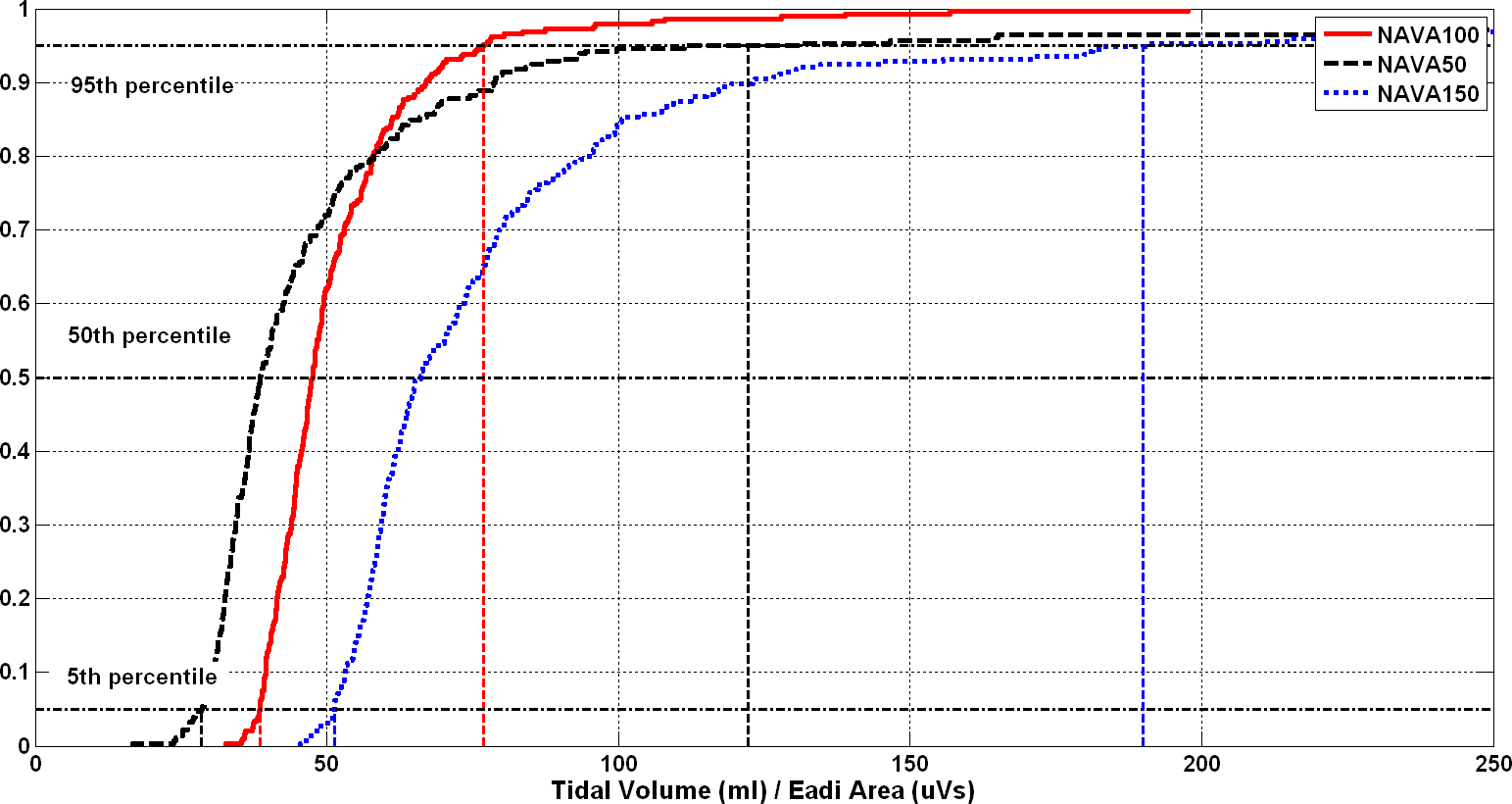
**

**Patient 3**

**
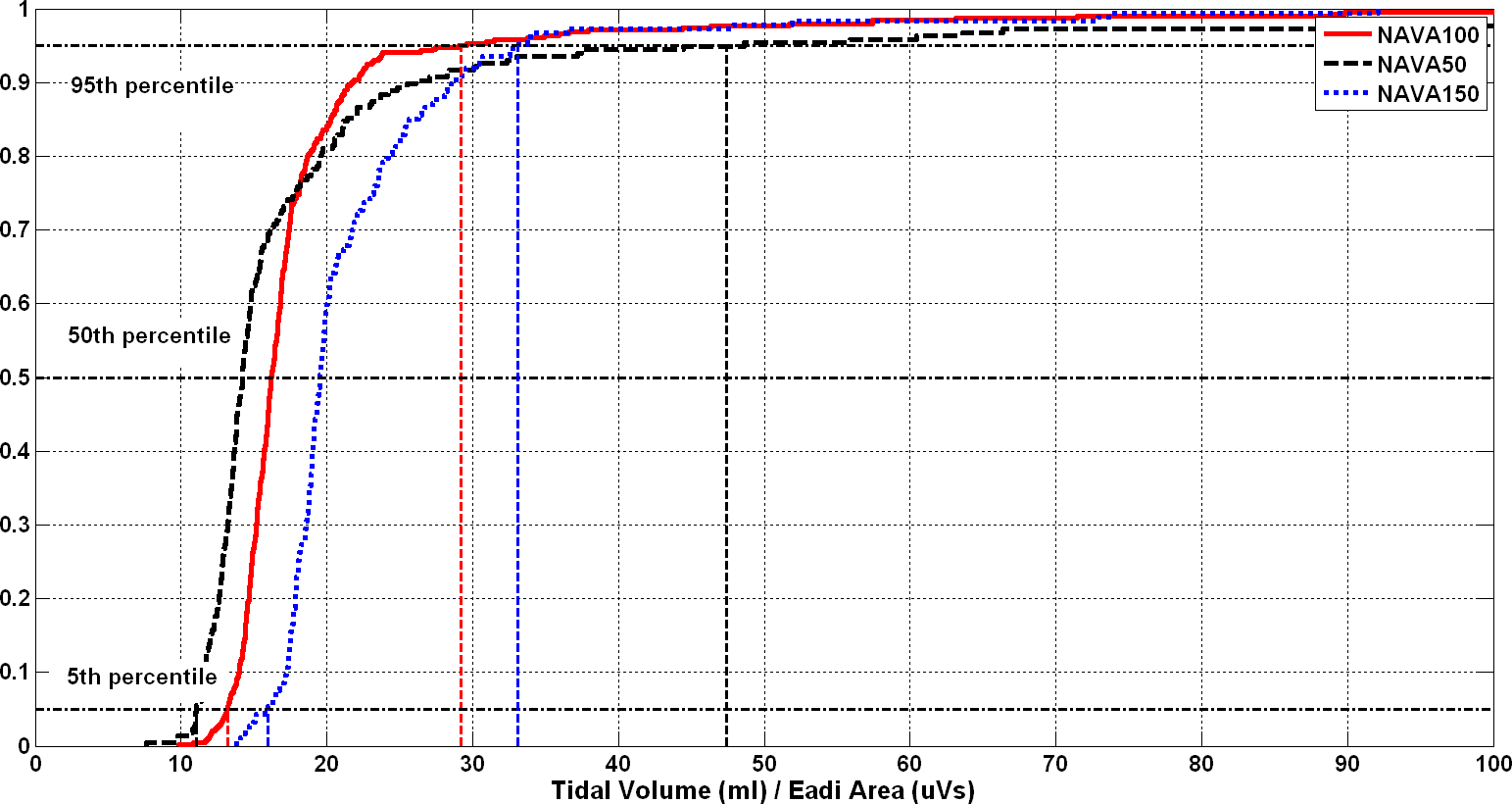
**

**Patient 4**

**
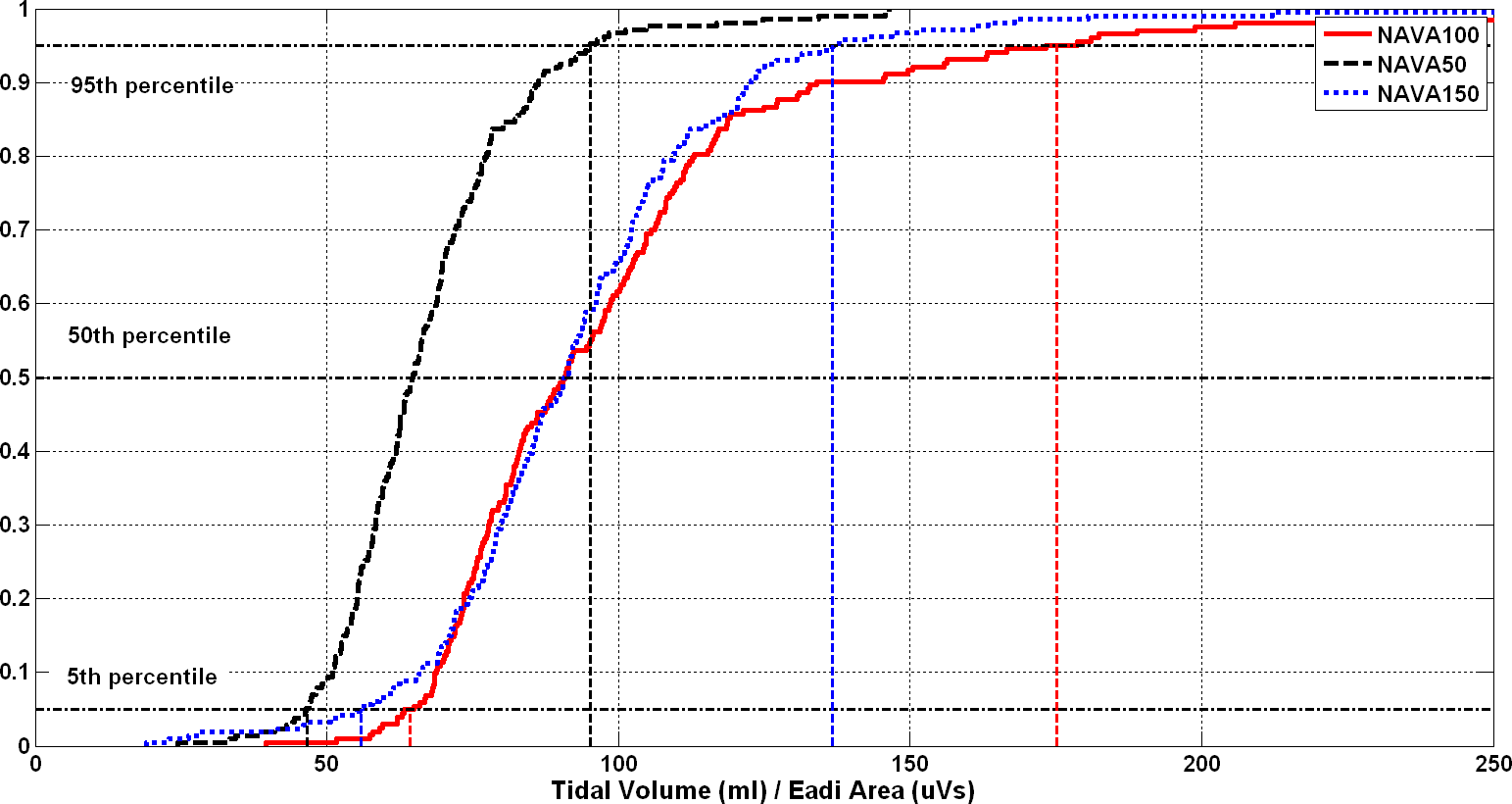
**

**Patient 5**

**
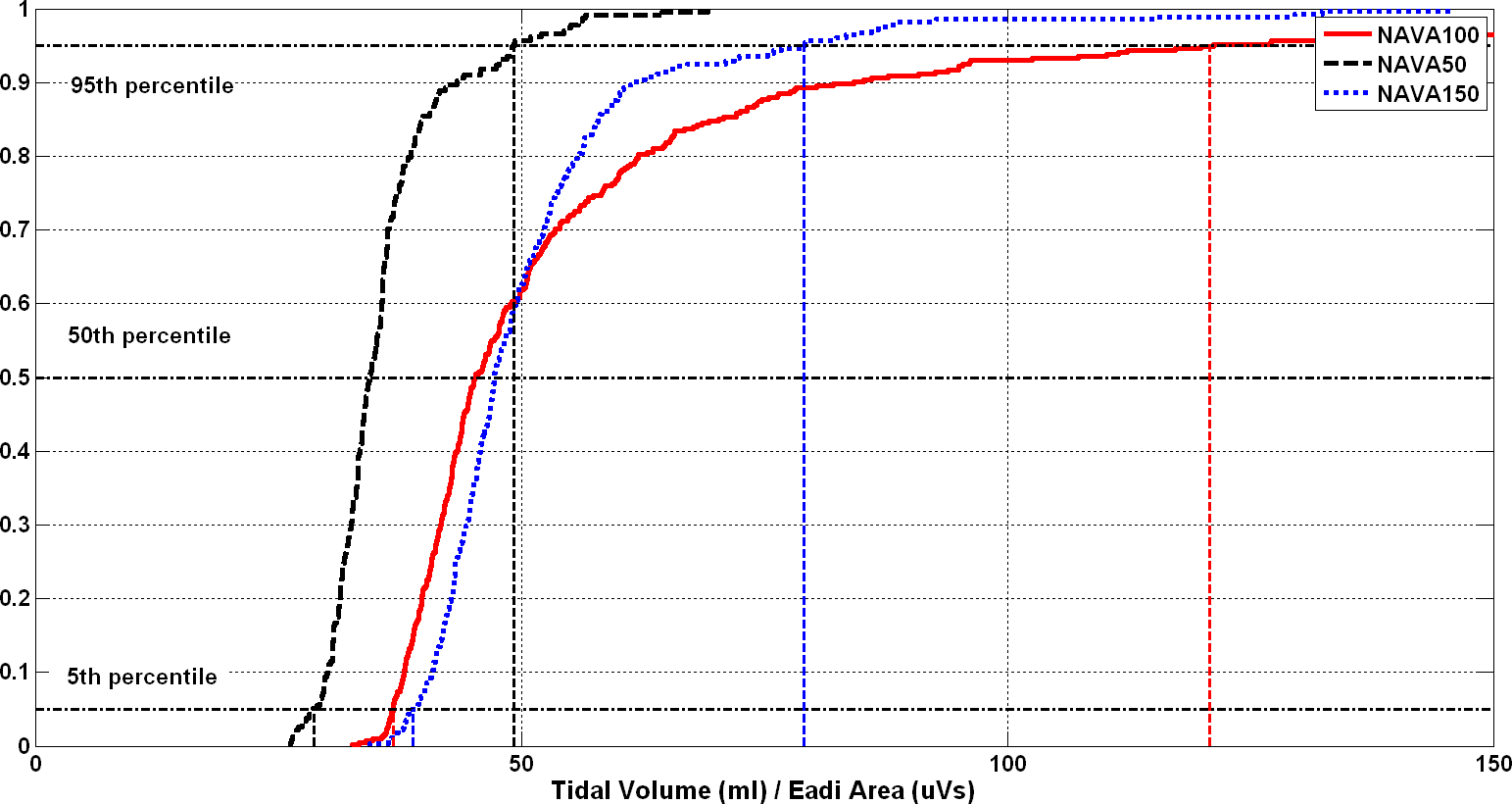
**

**Patient 6**

**
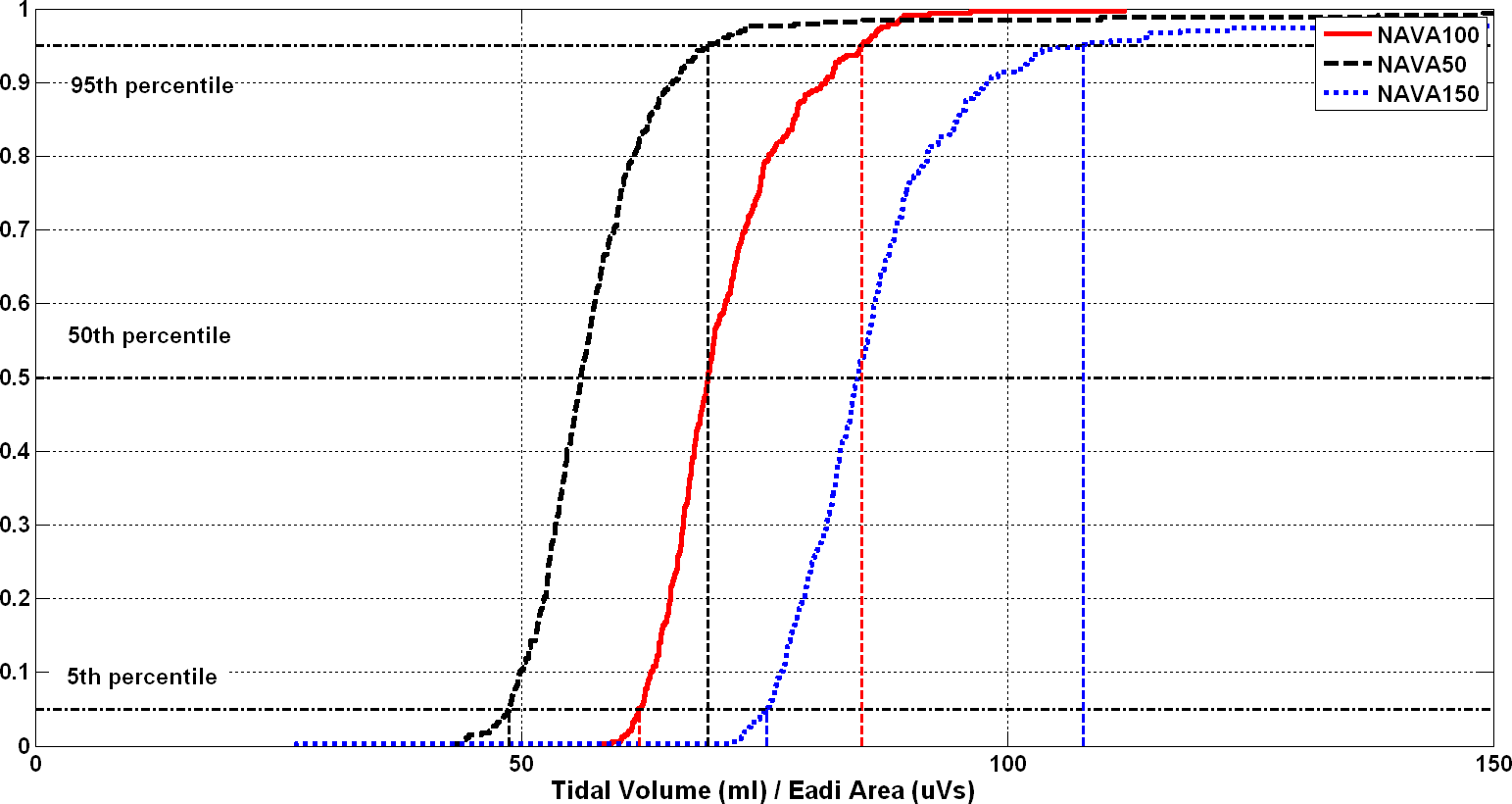
**

**Patient 7**

**
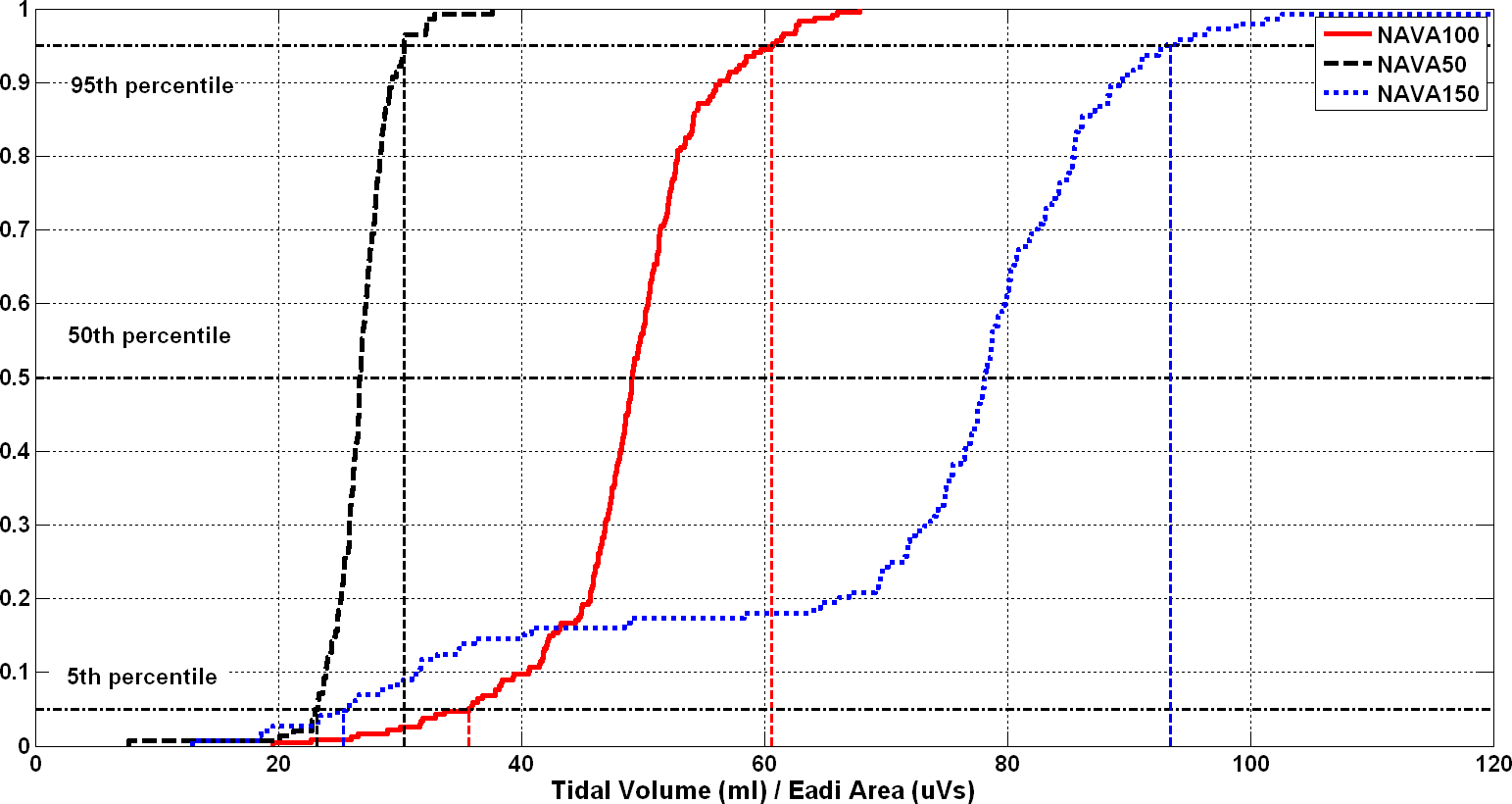
**

**Patient 8**

**
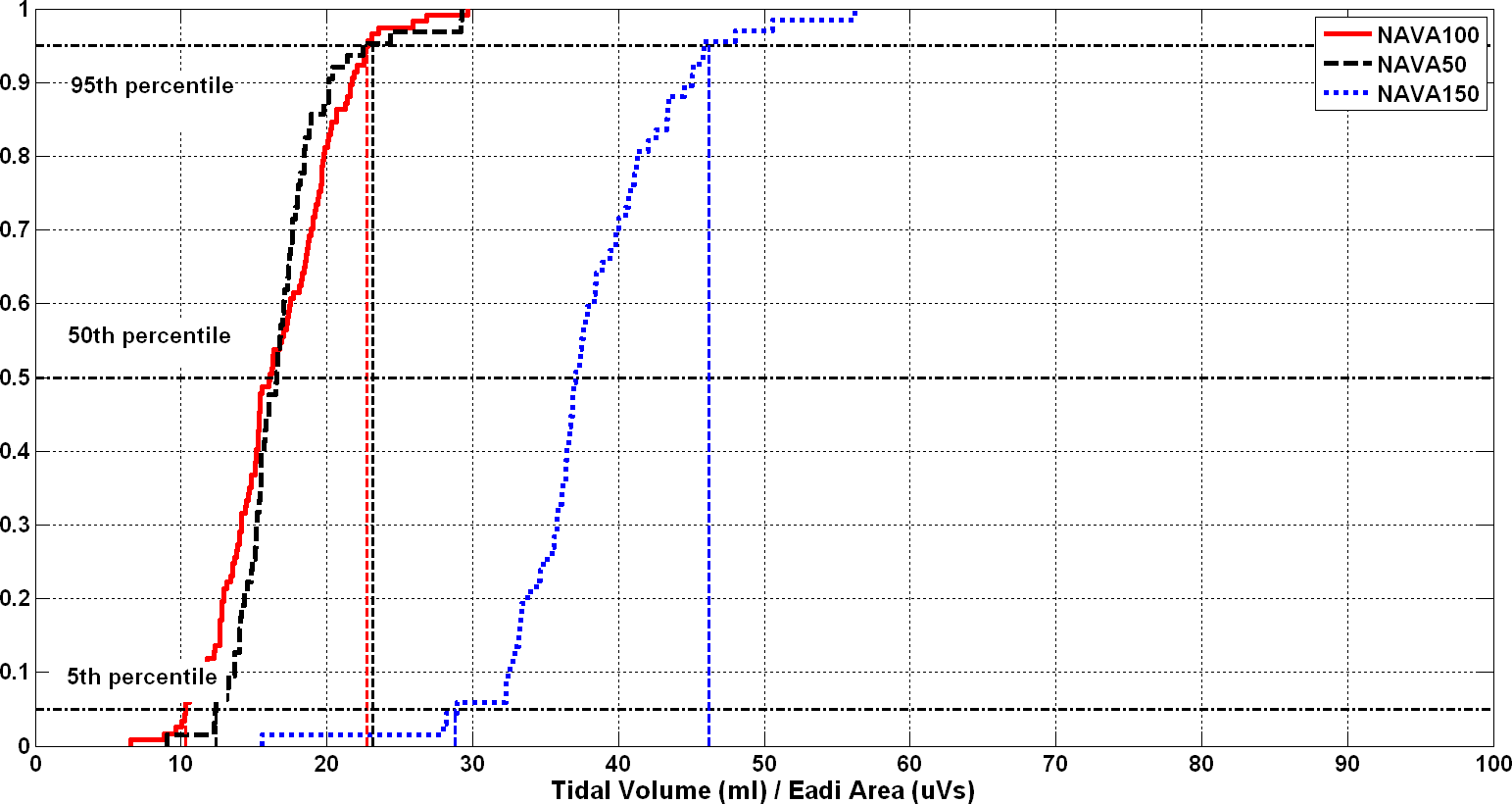
**

**Patient 9**

**
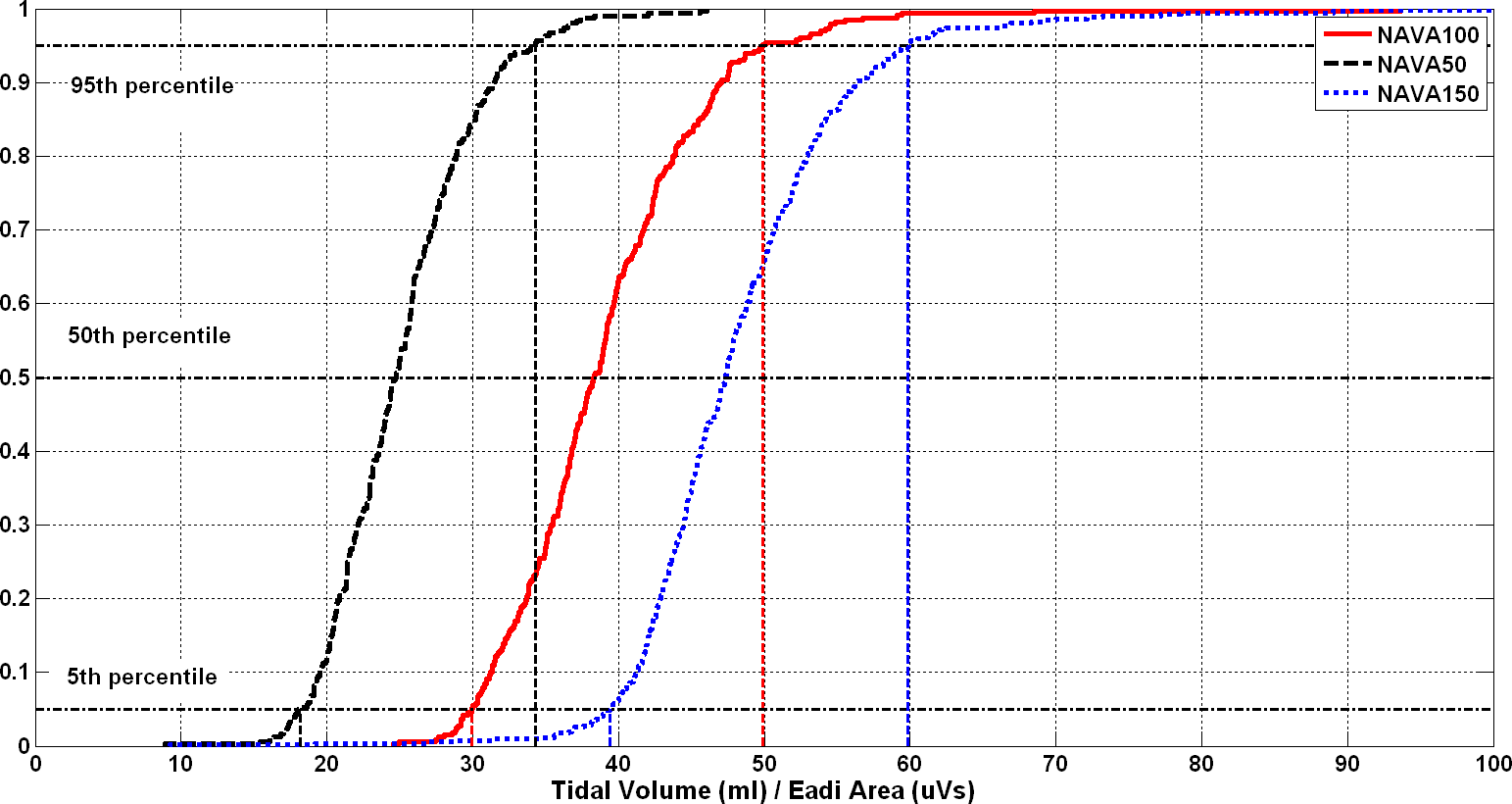
**

**Patient 10**

**
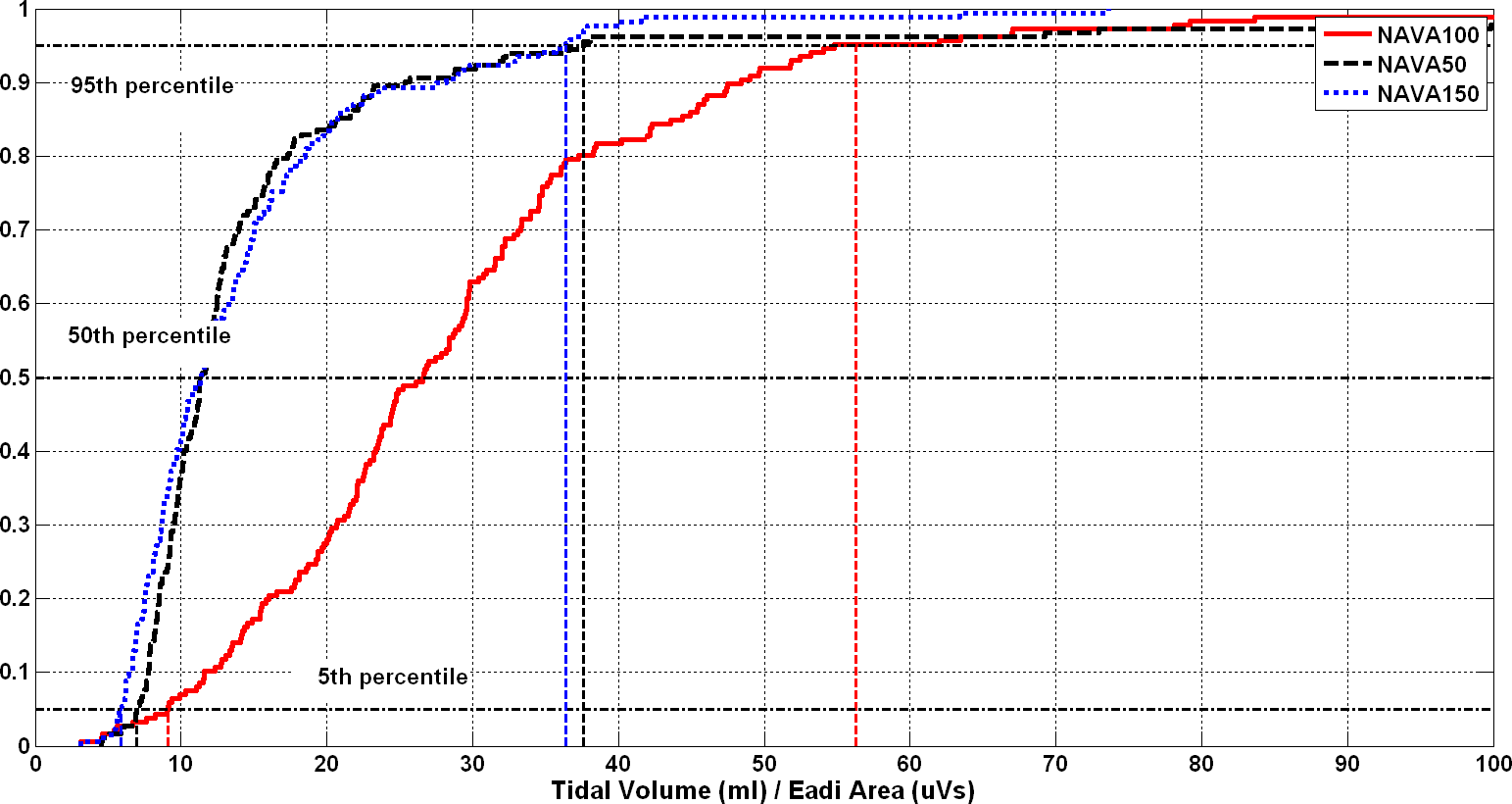
**

**Patient 11**

**
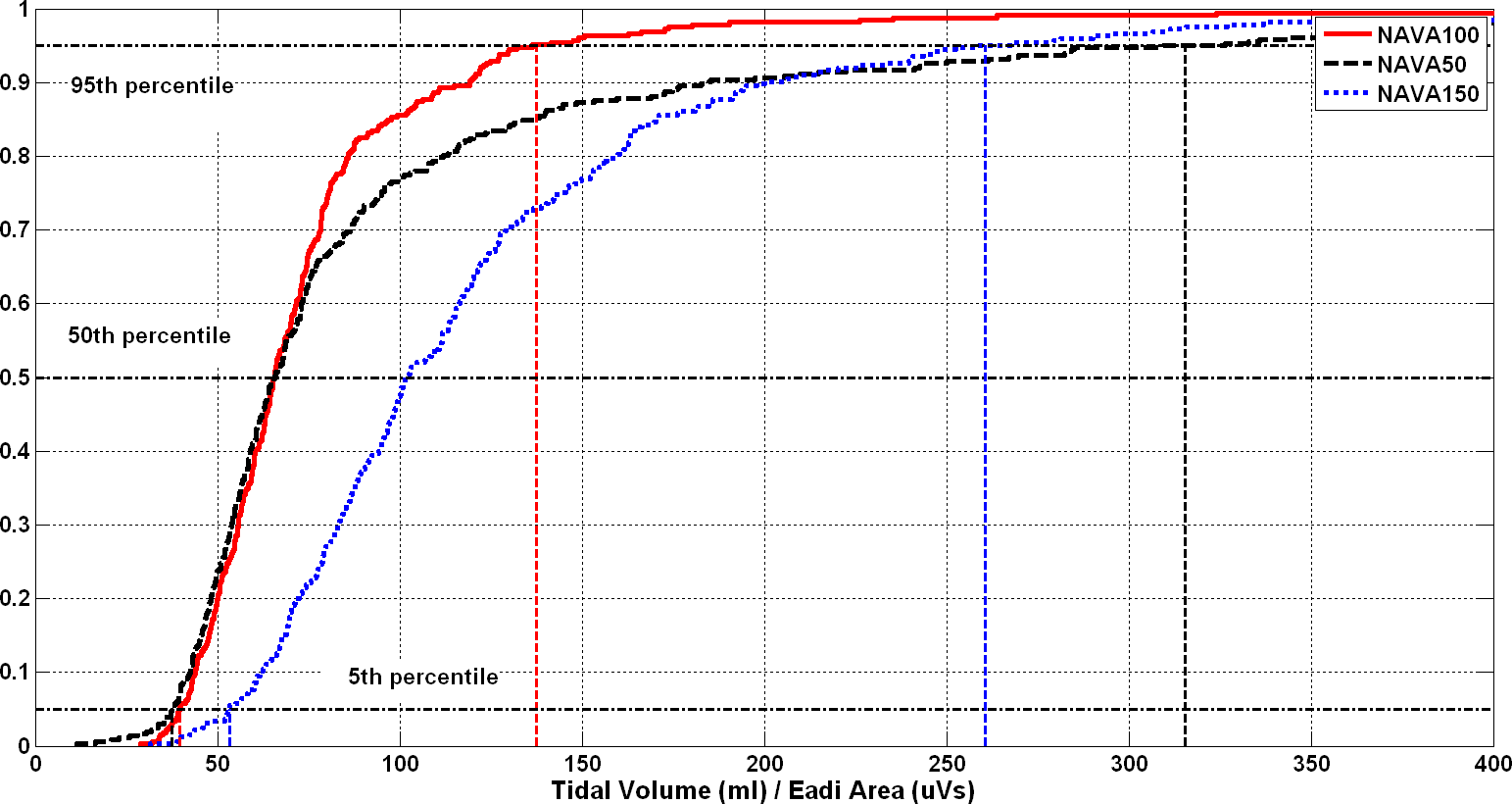
**

**Patient 12**

**
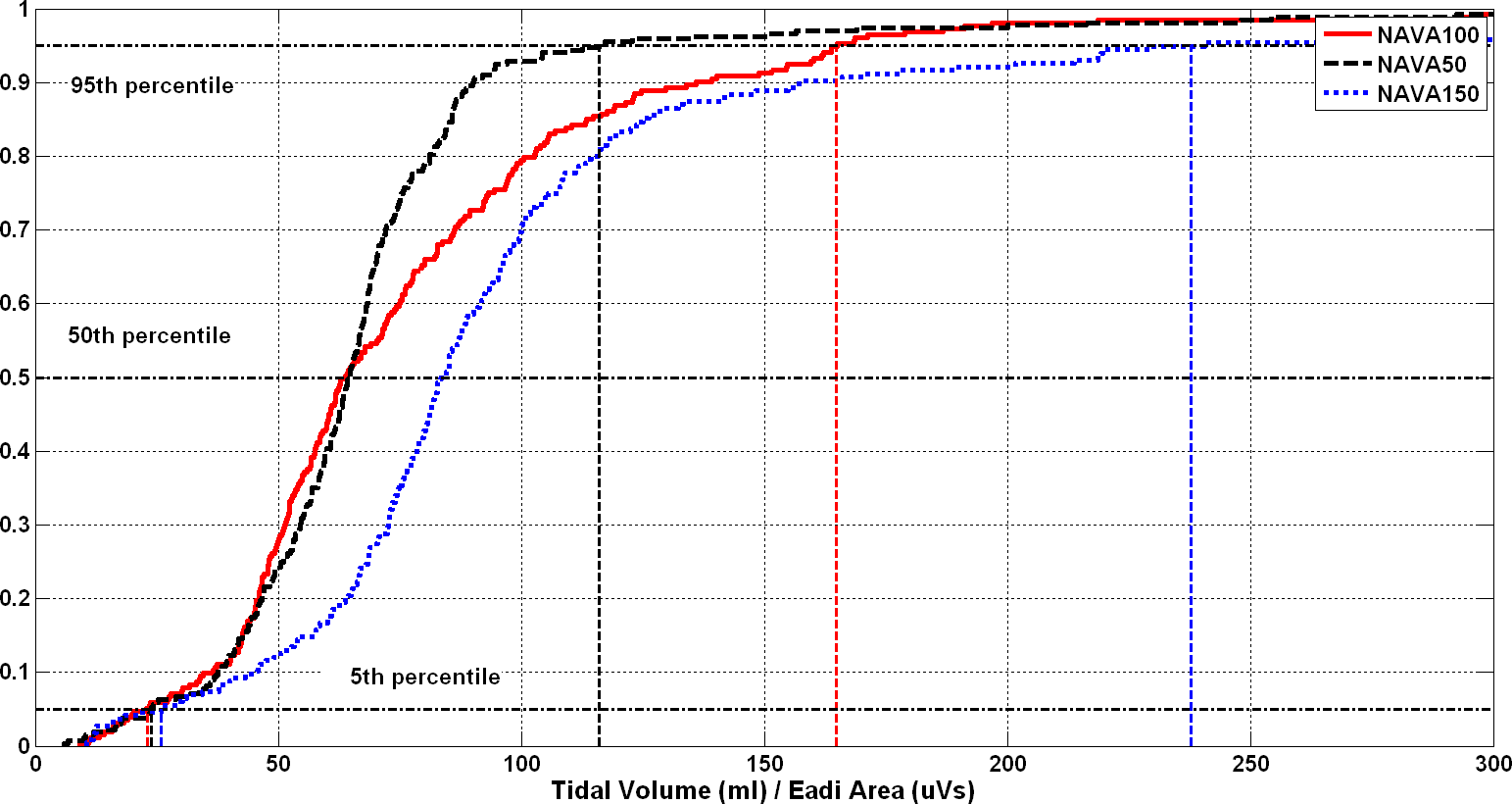
**
